# Falsification of the instrumental variable conditions in Mendelian randomization studies in the UK Biobank

**DOI:** 10.1007/s10654-023-01003-6

**Published:** 2023-05-31

**Authors:** Kelly Guo, Elizabeth W. Diemer, Jeremy A. Labrecque, Sonja A. Swanson

**Affiliations:** 1 Department of Epidemiology, Erasmus MC, Rotterdam, the Netherlands; 2 Department of Child and Adolescent Psychiatry, Erasmus MC, Rotterdam, the Netherlands; 3 Department of Epidemiology, Harvard T.H. Chan School of Public Health, Boston, USA; 4 CAUSALab, Harvard T.H. Chan School of Public Health, Boston, USA; 5 Department of Epidemiology, University of Pittsburgh School of Public Health, Pittsburgh, USA

**Keywords:** Falsification, Instrumental inequalities, Mendelian randomization, Coronary artery disease, Causal inference, UK Biobank

## Abstract

Mendelian randomization (MR) is an increasingly popular approach to estimating causal effects. Although the assumptions underlying MR cannot be verified, they imply certain constraints, the instrumental inequalities, which can be used to falsify the MR conditions. However, the instrumental inequalities are rarely applied in MR. We aimed to explore whether the instrumental inequalities could detect violations of the MR conditions in case studies analyzing the effect of commonly studied exposures on coronary artery disease risk.

Using 1077 single nucleotide polymorphisms (SNPs), we applied the instrumental inequalities to MR models for the effects of vitamin D concentration, alcohol consumption, C-reactive protein (CRP), triglycerides, high-density lipoprotein (HDL) cholesterol, and low-density lipoprotein (LDL) cholesterol on coronary artery disease in the UK Biobank. For their relevant exposure, we applied the instrumental inequalities to MR models proposing each SNP as an instrument individually, and to MR models proposing unweighted allele scores as an instrument. We did not identify any violations of the MR assumptions when proposing each SNP as an instrument individually. When proposing allele scores as instruments, we detected violations of the MR assumptions for 5 of 6 exposures.

Within our setting, this suggests the instrumental inequalities can be useful for identifying violations of the MR conditions when proposing multiple SNPs as instruments, but may be less useful in determining which SNPs are not instruments. This work demonstrates how incorporating the instrumental inequalities into MR analyses can help researchers to identify and mitigate potential bias.

**Supplementary Information:**

The online version contains supplementary material available at 10.1007/s10654-023-01003-6.

## Introduction

Mendelian randomization (MR) has become a popular approach for estimating causal effects and is increasingly popular as new large genetic databases become available [1, 2]. Like any causal inference approach using observational data, MR requires causal assumptions. In brief, these methods require that genetic variants are (i) associated with the exposure of interest, (ii) only cause the outcome via the exposure, and (iii) share no causes with the outcome. We will refer to these conditions collectively as the instrumental conditions. Notably, these conditions are sufficient only for sharp null testing and bounding; additional assumptions are necessary for point estimation [3].

Although instrumental conditions (ii) and (iii) are not verifiable, there are methods that can be used to falsify them. In particular, the instrumental conditions imply a set of mathematical constraints known as the instrumental inequalities, which, if violated, imply that the observed data distribution in a particular dataset is inconsistent with the instrumental conditions [4–7]. Investigators can thus use these instrumental inequalities as one approach for falsifying the instrumental conditions. Diemer et al. 2020 [8] presented a description of how to use the instrumental inequalities as well as an application of the instrumental inequalities in the setting of MR studies with multiple genetic variants proposed as instruments. A practical question remains: to what extent do the instrumental inequalities detect violations in analyses of commonly studied exposures or commonly used databases in MR? To investigate this, we applied the instrumental inequalities to a series of MR analyses of the effect of vitamin D concentration, alcohol consumption, C-reactive protein (CRP), triglycerides, high-density lipoprotein (HDL) cholesterol and low-density lipoprotein (LDL) cholesterol on coronary artery disease risk in the UK Biobank. These specific exposures were chosen as examples that are commonly studied and have been studied using MR in the UK Biobank, as well as to explore a range of exposure types (e.g., biomarkers and behaviors; categorical and continuous measures).

## Methods

### Study design

The UK Biobank is a large prospective study of 502,648 adults, aged 40–69, recruited from across the United Kingdom between 2006 and 2010. Details of the cohort, including recruitment, assessment procedures, and quality control have been described in detail elsewhere [9, 10]. The UK Biobank received ethical approval from the National Health Service North West Multi-centre Research Ethics Committee (Research Ethics Committee reference: 11/NW/0382) and all participants provided written, informed consent. For each exposure under study, we restricted the eligible population to individuals with complete data on that exposure, the outcome of coronary artery disease, and that exposure’s proposed genetic instruments, resulting in sample sizes between 424,978 and 486,195 individuals (see Supplementary Information: S4). While this complete case analysis may result in selection bias [11], it is consistent with some common approaches used in MR studies within the UK Biobank.

### Exposures

We selected 6 exposures whose relationships to cardiovascular disease have been previously studied using MR: vitamin D concentration, alcohol consumption, CRP, triglycerides, HDL-cholesterol, and LDL-cholesterol [12–27].

Vitamin D, triglyceride, CRP, HDL-, and LDL-cholesterol levels were measured in blood samples collected at either the initial assessment visit or a repeat assessment visit conducted between 2012 and 2013. Details of biomarker measurements and assay performance in UK Biobank have been described in detail elsewhere [28]. Briefly, vitamin D concentration was assessed based on total 25-hydroxyvitamin D (25(OH)D) levels measured using the Diasorin Liason, a chemiluminescent immunoassay. CRP levels were measured using an immunoturbidimetric assay in a Beckman Coulter analyzer (AU5800 Analyzer, Beckman Coulter, CA). Triglyceride concentrations were measured using an enzymatic analysis on said Beckman Coulter analyzer. HDL-cholesterol levels were measured by enzyme inhibition analysis, and LDL-cholesterol levels were measured using enzymatic protective selection analysis on said Beckman Coulter analyzer. Because the instrumental inequalities can only be used with categorical exposures [4, 5], all these exposures were categorized into deciles. Frequency of alcohol consumption was assessed based on self-report questionnaire. Participants were asked “About how often do you drink alcohol?” with response options “Never”, “Special occasions only”, “1 to 3 times a month”, “Once or twice a week”, “3 to 4 times a week”, or “Daily or almost daily”. If participants felt the answer varied, they were instructed to give an average over the past year. This exposure was categorized using these response categories.

### Outcome

Participant electronic health records, including International Classification of Disease (ICD-10) diagnosis codes and Office of Population and Censuses Surveys (OPCS-4) procedure codes, have been integrated into UK Biobank [29]. Additionally, patients were asked to report diagnoses of cardiovascular disease using questionnaires, which were subsequently checked during a verbal interview with a trained nurse. Participants were considered to have coronary artery disease if they had experienced angina pectoris, acute or subsequent myocardial infarction or other acute or chronic ischemic heart disease (ICD-10 I20X, I21X, I123X, I24X, I25.5, I25.6, I25.8, I25.9) or if they previously underwent coronary procedure (OPCS-4 K40, K41, K43, K46, K49, K75, K45, K50.1-3) (see Supplementary Information: S2 and S3).

### Genetic variants

In order to identify genetic variants that had previously been used in MR studies of these specific exposures within the UK Biobank, we conducted a systematic review of PubMed and the UK Biobank archive using the search term “Mendelian random*”, and each of the six exposures. Studies were eligible for inclusion in the review if they explicitly reported using an MR approach, studied either vitamin D, alcohol use, triglyceride levels, CRP, LDL-, or HDL-cholesterol as an exposure, and conducted the analysis using a UK Biobank sample. This resulted in 30 articles, of which 12 were rejected based on full text review. After review, 9 articles on vitamin D concentration, 3 articles on alcohol use, 2 articles on CRP, and 4 articles on lipoproteins (LDL-cholesterol, HDL-cholesterol, or triglycerides) met the criteria and were included in the review (see Supplementary Information: S1).

From these articles, we proposed single nucleotide polymorphisms (SNPs) as instruments for one of the six exposures if they had been proposed as instruments in at least one previous study. SNPs were not included if previous studies indicated that they were associated with another phenotype on a possibly pleiotropic pathway. In total, we proposed 1077 SNPs as instruments, including 15 SNPs as instruments for vitamin D concentration, 28 SNPs as instruments for alcohol consumption, 528 SNP as an instrument for CRP, 22 SNPs as instruments for triglyceride levels, 82 SNPs as instruments for HDL-cholesterol, and 402 SNPs as instruments for LDL-cholesterol (see Supplementary Information for an overview of the proposed instruments). We also constructed an unweighted categorical allele score for each exposure.

### Analysis

The properties and use of the instrumental inequalities have been described in detail in publications by Pearl, 1995 [4], Glymour et al., 2012 [30] and Diemer et al., 2020 [8]. Intuitively, by using an instrumental variable model, investigators are assuming that the observed data and unobserved counterfactuals from a population follow certain constraints (i.e., a particular ‘shape’). One way for investigators to check whether the instrumental variable assumptions hold would be to check whether the observed data fits within the ‘shape’ assumed by the instrumental variable model. The instrumental inequalities are simply a means of conducting this check, by evaluating whether a particular dataset conforms to these constraints.

More specifically, the instrumental inequalities are a set of mathematical equations derived from the instrumental conditions by Pearl, 1995 [4]:

$$\mathop {{\rm{max}}}\limits_x {\sum _y}\mathop {{\rm{max}}}\limits_z P\left( {x,y|z} \right) \le 1$$

where *Z* is the proposed instrument (or proposed instrument set), *X* is the exposure, and *Y* is the outcome. These equations evaluate whether proportions of certain combinations of the proposed instruments, exposure, and outcome sum to equal or less than one. If one or more of these inequalities do not hold, meaning that the sum of the observed probabilities is greater than one, it implies that at least one of the instrumental conditions is violated within the dataset. However, if the instrumental inequalities do hold, it does not constitute evidence for or against the instrumental conditions. Such a result does not imply that the instrumental conditions are satisfied within the specific dataset, only that we are unable to prove the instrumental conditions are violated in that dataset. Thus, the instrumental inequalities can be used to falsify (but not verify) an MR model. Moreover, the instrumental inequalities cannot show why the instrumental conditions are violated, only that they are violated. For MR studies, such violations can result from a number of different bias structures, including pleiotropy, selection bias, population stratification, and assortative mating [8, 31]. One key limitation of the instrumental inequalities is that they cannot be applied to continuous exposures [5]. While it is computationally easy to resolve this by discretizing the continuous exposures of interest, the instrumental conditions can then be violated by the measurement error induced by this categorization (sometimes known as coarsening) [31]. A simplified example demonstrating the application of the instrumental inequalities and the corresponding R code can be found in the Supplemental Information (see Supplemental Information: S6 and S11).

When multiple SNPs are proposed as instruments in MR, we can also apply the instrumental inequalities to sets of SNPs jointly [8]. For each exposure-outcome combination, we applied the instrumental inequalities to models proposing each SNP as an instrument individually and to a model proposing unweighted allele score deciles as an instrument, using R code developed in Diemer et al., 2020 [8] (see Supplementary Information: S12). As a sensitivity analysis to understand how results are impacted by residual population stratification, we also calculated the instrumental inequalities using inverse probability weights to adjust for 10 principal components (see Supplementary Information: S7). All analyses were conducted in R version 3.2.6 [32].

### Comparison to other falsification strategies: MR-Egger and MR-PRESSO

We also compared the results of the instrumental inequalities to the results of a MR-Egger intercept test and a MR Pleiotropy RESidual Sum and Outlier (PRESSO) global test. Both are commonly used falsification strategies in MR [33–35]. The MR-Egger method conducts an inverse variance weighted linear regression of the instrument-outcome association on the instrument-exposure association. If a test of the estimated intercept of this regression rejects the null hypothesis that the intercept is zero, then one often concludes that the instrumental conditions do not hold. The MR-PRESSO global test conducts multiple inverse variance weighed regressions to compute a residual sum of squares for each instrument in a set of proposed instruments, omitting one candidate instrument in each computation. It evaluates whether any of the proposed instruments are driving a difference between the total residual sum of squares and the simulated expected residual sum of squares. If the total residual sum of squares is inconsistent with what is expected by chance, the test rejects the null hypothesis of no horizontal pleiotropy; suggesting that the instrumental conditions are violated.

Unlike the instrumental inequalities, the MR-Egger and MR-PRESSO tests do not require us to coarsen continuous exposures. However, in addition to the instrumental conditions, they also evaluate whether linearity and homogeneity assumptions are satisfied [33–36]. A comparison of these falsification strategies’ assumptions, interpretation, and practical considerations are reviewed in Table 1. To compare the instrumental inequalities to the MR-Egger intercept test and MR-PRESSO, we applied both falsification methods to models proposing SNPs jointly as instruments for vitamin D concentration, alcohol consumption, CRP, triglyceride levels, HDL-cholesterol, and LDL-cholesterol.

**Table 1:** Overview of key assumptions, interpretations, and practical considerations of the instrumental inequalities, MR-Egger intercept test and MR-PRESSO global test

	Instrumental inequalities	MR-Egger intercept test	MR-PRESSO global test
Key assumptions in addition to the IV conditions	Coarsening of continuous exposures does not induce violations of IV conditions [31]	Homogeneity and linearity in associations between instrument, exposure, and outcome [34]	Homogeneity and linearity in associations between instrument, exposure, and outcome [35]
Example interpretations of the falsification test	(1) A global test of the IV conditions and the coarsening assumption (2) Assuming coarsening of continuous exposures did not induce violations, a test of the IV conditions	(1) A global test of the IV conditions, homogeneity, and linearity assumptions (2) Assuming homogeneity and linearity, a test of the IV conditions	(1) A global test of the IV conditions, homogeneity, and linearity assumptions (2) Assuming homogeneity and linearity, a test of the IV conditions
Considerations for statistical inference	No consensus currently exists on the optimal approach for statistical inference [40]	Often limited in power to be able detect violations of the instrumental conditions [38]	Possibly limited in power to detect violations of the instrumental conditions if the percentage of horizontal pleiotropic proposed instruments is less than 10%; less powerful to detect violations of the instrumental conditions when pleiotropy is imbalanced [35]
Applicability to one sample and/or two sample data settings	Presented approach only applicable in the one-sample setting	Has been applied in both one- and two-sample settings, but may produce biased results when applied to one sample setting [37]	Currently only applied to two-sample data settings

## Results

The study population in our analytic subpopulation consisted of participants with a median age of 58 years (ranging from 38 to 73 years) who were primarily of white, British ethnicity. The proportion of women varied between 53.5% and 54.2% across samples. A more detailed overview of the demographic and exposure characteristics of the study population can be found in the Supplementary Information (see Supplementary Information: S5).

The instrumental inequalities held for all 1077 SNPs proposed as instruments when considering each SNP individually (Table 2 and Supplementary Tables 2–7). The instrumental inequalities also held when allele scores were proposed as instruments for vitamin D. However, the instrumental inequalities were violated when proposing allele scores as instruments for alcohol consumption, CRP, triglycerides, HDL-cholesterol and LDL-cholesterol indicating that the instrumental conditions were violated for those models. Results were generally consistent when inverse probability weighted for 10 principal components (Supplementary Tables 8–13).

**Table 2:** Summary of results of instrumental inequalities applied to Mendelian randomization models studying the effects of vitamin D concentration, alcohol consumption, C-reactive protein, triglycerides, HDL-cholesterol, and LDL-cholesterol concentrations on coronary artery disease

Exposure	Number of proposed instruments	Number of violations of IV inequalities marginally	Maximum value of the inequalities for all SNPs marginally	Violation of IV inequalities for allele score	Maximum value of instrumental inequalities for allele score
Vitamin D	15	0/15	0.36	No	0.55
Alcohol consumption	28	0/28	0.34	Yes	1.11
CRP	528	0/528	0.26	Yes	1.25
Triglycerides	22	0/22	0.14	Yes	1.02
HDL-cholesterol	82	0/82	0.15	Yes	1.05
LDL-cholesterol	402	0/402	0.51	Yes	1.50

The instrumental inequalities were violated when proposing SNPs jointly as instruments for vitamin D, alcohol consumption, CRP, triglycerides and HDL-cholesterol and LDL-cholesterol levels (see Supplementary Information: S10). The MR-Egger intercept test showed a significant non-zero intercept when proposing SNPs jointly as instruments for CRP (intercept = 2.971 × 10^-4^, p < 0.001), triglyceride (intercept = 8.931 × 10^-4^, p < 0.001) and HDL-cholesterol levels (intercept = -3.474 × 10^-4^, p = 0.018), but not for vitamin D (intercept = 3.588 × 10^-5^, p = 0.914), alcohol consumption (intercept = 3.427 × 10^-4^, p = 0.081) and LDL-cholesterol levels (intercept = 5.560 × 10^-5^, p = 0.233). The MR-PRESSO global test was statistically significant for alcohol consumption, CRP, triglycerides, HDL-cholesterol, LDL-cholesterol, but not for vitamin D.

## Discussion

In our investigation of 6 exposures and an accompanying 1077 SNPs used in prior MR studies in UK Biobank, we detected no violations of the instrumental conditions when considering each SNP individually as a proposed instrument. Violations of the instrumental conditions were detected for some allele scores proposed as instruments.

These findings suggest that the instrumental inequalities may be helpful in detecting violations of the instrumental conditions for sets of SNPs proposed as instruments, but, per prior conventional wisdom, may not detect which specific SNP or SNPs are not instruments. Moreover, these findings do not explain why the allele scores are not instruments: we do not know if violations of the instrumental conditions are due to selection bias, pleiotropy or due to another structural violation. It also remains unknown if the violation is a result of one or multiple SNPs marginally violating the instrumental conditions. Additionally, these findings do not tell us whether the violation is study-specific or could indicate a structural violation with that allele score being an instrument for that exposure-outcome in another study setting [8]. It is worth reiterating that detecting no violations when considering each SNP individually should only be interpreted as a failure to falsify, and not as support for the validity. It is possible that the instrumental conditions are violated for all SNP, exposure, and outcome combinations in this study, but the instrumental inequalities detected only some of these violations. Our results also need to be interpreted in light of the imposed categories of the continuous exposures [8].

Notably, the results of the instrumental inequalities, MR-Egger and MR-PRESSO diverged to some extent for several of the exposures of interest. In MR models for the effect of CRP, triglycerides, and HDL-cholesterol on coronary artery disease, violations of the instrumental conditions were detected by the instrumental inequalities, MR-Egger, and by MR-PRESSO when proposing all SNPs jointly as instruments. Violations were also detected by both MR-PRESSO and the instrumental inequalities when proposing all SNPs jointly as instruments for alcohol consumption and LDL-cholesterol, though MR-Egger did not detect any violations of the instrumental conditions for these exposures. MR-Egger and MR-PRESSO also did not detect any violations for vitamin D and LDL-cholesterol, though the instrumental inequalities were violated for both when proposing all SNPs jointly as instruments, and also when proposing an allele score as an instrument for LDL-cholesterol.

These differences suggest that these falsification strategies can be complementary to one another, as there are a number of possible reasons why the results of these three methods may diverge. First, some research has indicated that MR-Egger may produce biased results when applied to single-sample applications [37], and the MR-Egger intercept test may often be underpowered to detect violations of the MR assumptions [38]. Second, as discussed earlier, the inequalities require categorization of the exposure, meaning violations of the inequalities may indicate bias resulting from said categorization, while the MR-Egger intercept test and MR-PRESSO global test require additional linearity and homogeneity assumptions that are not guaranteed to hold in this setting. In addition, previous work has suggested that, as the number of proposed joint instruments grows and the number of individuals within strata of the proposed joint instrument decreases, the instrumental inequalities may become increasingly sensitive to random violations of the MR conditions [8]. Overall, our findings suggest that further work is needed to evaluate the relative sensitivity of the instrumental inequalities, MR-Egger intercept test and MR-PRESSO global test to different forms of bias, but that these three methods appear to provide information about the validity of the MR assumptions in applied studies.

MR studies rely on strong, unverifiable assumptions, but investigators have an arsenal of tools for falsifying these assumptions and attempting to mitigate violations, along with robust methods that leverage alternative assumptions as a means to relax some others [34, 39]. The instrumental inequalities are an easily implementable technique, which, if integrated into this MR toolbox, could help to identify violations of the instrumental conditions in common MR settings with multiple proposed instruments, including biases that may be difficult to identify through other means.

## Electronic supplementary material

Below is the link to the electronic supplementary material.

Supplementary Material 1

## Data Availability

The data are available from the UK Biobank to individuals who apply for and receive permission from the appropriate UK Biobank study committees.
