# Supplementary material for: Falsification of the instrumental variable conditions in Mendelian randomization studies in the UK Biobank
Source: Eur J Epidemiol. 2023 May 31;38(9):921–7. doi: 10.1007/s10654-023-01003-6 (PMC10501946; doi:10.1007/s10654-023-01003-6)
Supplement: Supplementary file 1 — Supplementary Material 1 [file 10654_2023_1003_MOESM1_ESM.docx]

Supplementary Information for:

**Falsification of the instrumental variable conditions in Mendelian randomization studies in the UK Biobank**

Kelly Guo^1*^, Elizabeth W. Diemer^2, 3, 4^, Jeremy A. Labrecque^1^, & Sonja A. Swanson^1, 3, 4, 5^

1. Department of Epidemiology, Erasmus MC, Rotterdam, the Netherlands 2. Department of Child and Adolescent Psychiatry, Erasmus MC, Rotterdam, the Netherlands 3. Department of Epidemiology, Harvard T.H. Chan School of Public Health, Boston, USA, 4. CAUSALab, Harvard T.H. Chan School of Public Health, Boston, USA, 5. Department of Epidemiology, University of Pittsburgh School of Public Health, Pittsburgh, USA

*Corresponding author: Tel: +31107043488, Fax: +31107044657, Email: k.guo@erasmusmc.nl

**Supplementary content:**

# S1. Details of systematic review to identify commonly proposed genetic instruments

# S2. ICD-10 and OPCS-4 coding and descriptions

# S3. Data-Fields in UK Biobank

# S4. Supplementary figures 1-6: Flowcharts showing inclusion and exclusion of participants

# S5. Supplementary table 1: Demographic and exposure characteristics of study population

# S6. Toy example

# S7. Details of inverse probability weighting procedure

# S8. Supplementary tables 2-7: Results of the instrumental inequalities for each SNP proposed as an instrument

# S9. Supplementary tables 8-13: Results of the instrumental inequalities for each SNP proposed as an instrument in inverse-probability weighted pseudo populations

# S10. Supplementary table 13: Results of the instrumental inequalities, MR-Egger intercept test and MR-PRESSO global test for SNPs proposed jointly instruments

# S11. R Code: Simulating a toy example for the instrumental inequalities

# S12. R Code: Computing the maximum value of the instrumental inequalities for a single joint instrument

**S1. Details of systematic review to identify commonly proposed genetic instruments**

To identify SNPs commonly proposed as instruments for each exposure, we searched the UK Biobank archive (https://www.ukbiobank.ac.uk) and PubMed and the using the following search terms:

| **Vitamin D** | Mendelian random*[tiab] AND (UK Biobank[tiab] OR UK-biobank[tiab]) AND (“Vitamin D”[Mesh] OR “25-Hydroxyvitamin D 2”[Mesh]) |
| --- | --- |
| **Alcohol** | Mendelian random*[tiab]) AND (UK Biobank[tiab] OR UK-biobank[tiab]) AND (“Drinking Behavior”[Mesh]) |
| **CRP** | Mendelian random*[tiab] AND (UK Biobank[tiab] OR UK-biobank[tiab]) AND (“C-Reactive Protein”[Mesh]) |
| **Lipoprotein (LDL-cholesterol, HDL-cholesterol, triglycerides)** | Mendelian random*[tiab] AND (UK Biobank[tiab] OR UK-biobank[tiab]) AND (“Triglycerides”[Mesh] OR “Cholesterol, HDL”[Mesh] OR “Cholesterol, LDL” [Mesh]) |

Databases were searched from their start date to March 2020. Initial searches resulted in 30 potentially eligible studies. Studies were eligible for inclusion in the review if they (1) explicitly reported using an MR approach, (2) studied either vitamin D, alcohol use, triglyceride levels, CRP, LDL-, or HDL-cholesterol as an exposure, and (3) conducted the analysis within a UK Biobank sample. Importantly, studies were not required to use coronary artery disease as an outcome. Of the 30 articles, 12 were rejected based on full text review. After review, 9 articles on vitamin D concentration, 3 articles on alcohol use, 2 articles on CRP, and 4 articles on lipoproteins (LDL-cholesterol, HDL-cholesterol, or triglycerides) met criteria and were included in the review. A list of included articles and SNPs proposed in each is available below.

| **Instruments proposed in UK Biobank studies (all exposures)** | | | |
| --- | --- | --- | --- |
| *Title* | *Reference* | *Year* | *Proposed Instruments* |
| **Vitamin D** |  |  |  |
| *Vitamin D levels and susceptibility to asthma, elevated immunoglobulin E levels, and atopic dermatitis: A Mendelian randomization study* | Manousaki D, Paternoster L, Standl M, et al. Vitamin D levels and susceptibility to asthma, elevated immunoglobulin E levels, and atopic dermatitis: A Mendelian randomization study. *PLoS Med*. 2017;14(5):e1002294. | 2017 | rs2282679  rs12785878  rs10741657  rs6013897 |
| *Vitamin D and cognitive function: A Mendelian randomisation study* | Maddock J, Zhou A, Cavadino A, et al. Vitamin D and cognitive function: A Mendelian randomisation study. *Sci Rep*. 2017;7(1):13230. | 2017 | rs12785878 rs12794714 |
| *Vitamin D and overall cancer risk and cancer mortality: a Mendelian randomization study* | Ong JS, Gharahkhani P, An J, et al. Vitamin D and overall cancer risk and cancer mortality: a Mendelian randomization study. Hum Mol Genet. 2018;27(24):4315-4322. | 2018 | rs12785878  rs116970203  rs10741657  rs6013897  rs2282679 |
| *Vitamin D and Inflammatory Bowel Disease: Mendelian Randomization Analyses in the Copenhagen Studies and UK Biobank.* | Lund-Nielsen J, Vedel-Krogh S, Kobylecki CJ, Brynskov J, Afzal S, Nordestgaard BG. Vitamin D and Inflammatory Bowel Disease: Mendelian Randomization Analyses in the Copenhagen Studies and UK Biobank. *J Clin Endocrinol Metab*. 2018;103(9):3267-3277. | 2018 | rs7944926 rs11234027 rs10741657  rs12794714 |
| *Exploring causality in the association between circulating 25-hydroxyvitamin D and colorectal cancer risk: a large Mendelian randomisation study* | He Y, Timofeeva M, Farrington SM, et al. Exploring causality in the association between circulating 25-hydroxyvitamin D and colorectal cancer risk: a large Mendelian randomisation study. *BMC Med*. 2018;16(1):142. | 2018 | rs3755967 rs10741657 rs12785878 rs17216707 rs10745742 rs8018720 |
| *Circulating Serum 25-Hydroxyvitamin D Levels and Bone Mineral Density: Mendelian Randomization Study* | Larsson SC, Melhus H, Michaëlsson K. Circulating Serum 25-Hydroxyvitamin D Levels and Bone Mineral Density: Mendelian Randomization Study. *J Bone Miner Res*.  2018;33(5):840-844 | 2018 | rs2282679  rs117913124 rs10741657  rs12785878  rs6013897 |
| *Assessing the causal association between 25-hydroxyvitamin D and the risk of oral and oropharyngeal cancer using Mendelian randomization* | Dudding T, Johansson M, Thomas SJ, Brennan P, Martin RM, Timpson NJ. Assessing the causal association between 25-hydroxyvitamin D and the risk of oral and oropharyngeal cancer using Mendelian randomization. *Int J Cancer*. 2018;143(5):1029-1036. | 2018 | rs4588  rs116970203  rs4423214  rs10741657  rs6013897 |
| *Association of maternal circulating 25(OH)D and calcium with birth weight: A mendelian randomisation analysis* | Thompson WD, Tyrrell J, Borges MC, et al. Association of maternal circulating 25(OH)D and calcium with birth weight: A mendelian randomisation analysis. *PLoS Med*. 2019;16(6):e1002828. | 2019 | rs10741657  rs117913124  rs12785878 rs3755967  rs17216707  rs10745742 rs8018720 |
| *Investigating causality in the association between vitamin D status and self-reported tiredness* | Havdahl A, Mitchell R, Paternoster L, Davey Smith G. Investigating causality in the association between vitamin D status and self-reported tiredness. Sci Rep. 2019;9(1):2880. | 2019 | rs3755967 rs12785878 rs10741657  rs17216707  rs117913124  rs8018720  rs10745742 |
| **Alcohol Use** |  |  |  |
| *Is There Causal Relationship of Smoking and Alcohol Consumption with Bone Mineral Density? A Mendelian Randomization Study* | Guo R, Wu L, Fu Q. Is There Causal Relationship of Smoking and Alcohol Consumption with Bone Mineral Density? A Mendelian Randomization Study. *Calcif Tissue Int*. 2018;103(5):546-553. | 2018 | rs145452708  rs193099203  rs1260326 rs11940694  rs29001570 rs9841829 |
| *Alcohol intake and risk of rheumatoid arthritis: a Mendelian randomization study* | Bae SC, Lee YH. Alcohol intake and risk of rheumatoid arthritis: a Mendelian randomization study. Alkoholkonsum und Risiko der rheumatoiden Arthritis: eine Mendel-Randomisierungsstudie. Z Rheumatol. 2019;78(8):791-796. | 2019 | rs11039429  rs11787216  rs11940694  rs1260326  rs13102973 rs13231886  rs13390019  rs17097556  rs17690703  rs1788030  rs1893659  rs2159935  rs363096  rs4726481  rs540606  rs571312  rs58905411  rs6016781  rs650558  rs7428430  rs838145  rs9372625  rs9842406  rs9923768 |
| *What is the effect of alcohol consumption on the risk of chronic widespread pain? A Mendelian randomisation study using UK Biobank* | Beasley M, Freidin MB, Basu N, Williams FMK, Macfarlane GJ. What is the effect of alcohol consumption on the risk of chronic widespread pain? A Mendelian randomisation study using UK Biobank. Pain. 2019;160(2):501-507. | 2019 | rs1229984 |
| **CRP** |  |  |  |
| *Causal Factors for Knee, Hip, and Hand Osteoarthritis: A Mendelian Randomization Study in the UK Biobank* | Funck-Brentano T, Nethander M, Movérare-Skrtic S, Richette P, Ohlsson C. Causal Factors for Knee, Hip, and Hand Osteoarthritis: A Mendelian Randomization Study in the UK Biobank. *Arthritis Rheumatol*. 2019;71(10):1634-1641. | 2019 | rs4420065  rs4129267  rs2794520  rs12239046  rs6734238  rs4705952  rs6901250  rs9987289  rs1183910  rs10521222  rs2847281  rs1800961 |
| *Using Mendelian randomization to evaluate the causal relationship between serum C-reactive protein levels and age-related macular degeneration* | Han X, Ong JS, An J, Hewitt AW, Gharahkhani P, MacGregor S. Using Mendelian randomization to evaluate the causal relationship between serum C-reactive protein levels and age-related macular degeneration. Eur J Epidemiol. 2020;35(2):139-146. | 2020 | rs429358  rs7551731  rs7970695  rs2154384  rs61812598  rs1260326  rs17616063  rs6698653  rs80272044  rs4255379  rs116971887  rs7012637  rs55709272  rs190712692  rs12077265  rs1800961  rs56015600  rs79722469  rs141737681  rs16842320  rs144970957  rs2239222  rs728538  rs77056528  rs6519133  rs34415150  rs340005  rs28929474  rs1490384  rs2393794  rs2836882  rs117264457  rs3027003  rs469882  rs75460349  rs150852956  rs148303016  rs4767938  rs6486122  rs10095930  rs62118504  rs6501207  rs148933445  rs75995782  rs12231235  rs1037170  rs1412444  rs3768321  rs114451718  rs9266230  rs2965164  rs204914  rs72654472  rs150844304  rs1880241  rs3794204  rs6073958  rs7008413  rs635634  rs55855238  rs72959041  rs2269434  rs28399607  rs2700938  rs601338  rs146342974  rs2161037  rs9388766  rs1736057  rs61542988  rs56094641  rs2542170  rs687339  rs148770227  rs41290108  rs1056441  rs1441169  rs9366639  rs4871582  rs33951980  rs34061534  rs8178824  rs139136389  rs114733917  rs115273594  rs58542926  rs1292067  rs77120325  rs34874378  rs2681780  rs16835819  rs144398877  rs28601761  rs77719426  rs1800693  rs6961634  rs61821567  rs10203386  rs1570868  rs77704739  rs10849773  rs182638776  rs8009347  rs2269841  rs1476698  rs12141189  rs72743115  rs12448756  rs10083137  rs56189574  rs12426126  rs4266763  rs8060025  rs3750310  rs10752652  rs704017  rs9988620  rs1933736  rs55981844  rs663015  rs3865444  rs1800919  rs8040040  rs3808348  rs6265  rs12714415  rs11065050  rs4766960  rs76516194  rs3856270  rs2927447  rs6920220  rs144314390  rs8054651  rs12055445  rs9611454  rs7303035  rs143273561  rs78620885  rs6501199  rs2201637  rs6786055  rs2638315  rs36049560  rs79101008  rs4641306  rs2627646  rs4764939  rs2161374  rs1905505  rs116073407  rs846879  rs117368206  rs1545536  rs2207132  rs2068888  rs185575165  rs9738365  rs11246574  rs11648192  rs2250010  rs7189954  rs1985157  rs10760691  rs4411129  rs6905544  rs183553104  rs7084062  rs12132412  rs111237020  rs2980512  rs78912080  rs11229252  rs72694393  rs10755296  rs12933292  rs6020459  rs2239704  rs117211511  rs59059615  rs6792725  rs4714508  rs4755720  rs645692  rs41523449  rs3733892  rs11577023  rs62491814  rs148059420  rs11227395  rs111435864  rs1801272  rs11666245  rs17138476  rs2110944  rs78769612  rs7958316  rs6138537  rs7280982  rs142603322  rs2721953  rs1353792  rs149523036  rs3751143  rs8109532  rs4018180  rs73577882  rs11672020  rs385417  rs11078597  rs112689575  rs7502409  rs55665939  rs8077859  rs420957  rs2740479  rs6509155  rs4782568  rs116424768  rs10864086  rs150365073  rs1532085  rs72732974  rs2054434  rs12211604  rs8126001  rs35860194  rs12357890  rs623011  rs10802496  rs6962836  rs74085345  rs8059619  rs6845703  rs4939034  rs73137144  rs7956514  rs7528419  rs2265189  rs7319102  rs1800973  rs2301275  rs4148155  rs11057214  rs12138486  rs117695016  rs12620844  rs2131925  rs150543164  rs117548270  rs75497300  rs13106834  rs62389532  rs10106298  rs9383643  rs4535048  rs3774063  rs11693537  rs62011286  rs75898076  rs62106258  rs10924372  rs4239504  rs11000760  rs178835  rs74949966  rs17308476  rs11210869  rs1052067  rs7549881  rs148104667  rs58287327  rs12516176  rs11681145  rs13081778  rs17626434  rs1338721  rs111570031  rs12992747  rs35881303  rs2957664  rs1635852  rs6509222  rs8978  rs13066686  rs12425953  rs6072279  rs7993752  rs2668244  rs10810455  rs77101145  rs62282070  rs76102184  rs12735458  rs4871827  rs7102088  rs139952834  rs62513191  rs141651106  rs34284056  rs145987962  rs654912  rs8034216  rs123698  rs149647262  rs10848035  rs77243303  rs3746778  rs4915287  rs117803005  rs4084164  rs2432195  rs174373  rs11928797  rs184334219  rs787488  rs150377363  rs6433282  rs11667234  rs71658797  rs1886008  rs3125326  rs78703482  rs3789988  rs3826559  rs4865540  rs4704780  rs4788867  rs684016  rs245768  rs12716871  rs13108218  rs12992995  rs2030291  rs1495745  rs1693456  rs35764600  rs141094656  rs4431051  rs1969026  rs13058527  rs11708067  rs4849147  rs2011689  rs2256027  rs1975190  rs117849038  rs115462819  rs74354286  rs11158975  rs10831676  rs2882485  rs2075803  rs78343493  rs17854914  rs56960368  rs12966114  rs139460294  rs7537072  rs543874  rs3014201  rs674833  rs4876992  rs6595549  rs17417252  rs1441043  rs424539  rs209474  rs117783785  rs13416992  rs4658403  rs77151304  rs62121122  rs12679106  rs6816467  rs73201521  rs11012732  rs12466485  rs2233278  rs301804  rs2961111  rs72660319  rs4506495  rs12712928  rs11693697  rs12443906  rs78948780  rs62618693  rs114574084  rs11047139  rs12601665  rs11711864  rs10806385  rs2970901  rs34761529  rs6445393  rs112750178  rs115178836  rs68100606  rs17366139  rs3736164  rs17105232  rs12280456  rs263013  rs77808205  rs11083166  rs2107135  rs59316512  rs1492914  rs10490871  rs6840517  rs3785568  rs8008748  rs71414197  rs4499304  rs112283362  rs11767338  rs114947103  rs7430523  rs13227497  rs75064168  rs6443429  rs10779035  rs3134133  rs2302758  rs12828693  rs984181  rs6705820  rs12517168  rs288183  rs9826984  rs6090103  rs653170  rs769662  rs151132887  rs77522  rs7442885  rs71322200  rs6447335  rs61741874  rs62129471  rs2494097  rs613872  rs6591188  rs7171864  rs2808474  rs72837690  rs11652684  rs58770366  rs115093996  rs1290898  rs480958  rs157512  rs180686303  rs11772703  rs11648261  rs59737437  rs146396094  rs2472682  rs11231161  rs6984551  rs62204977  rs11222703  rs116804861  rs7241918  rs9368503  rs55824262  rs10794644  rs6595968  rs380825  rs62451586  rs35582280  rs62092069  rs3095122  rs10453441  rs28361325  rs1968109  rs1045075  rs10957159  rs57550938  rs1495017  rs11682186  rs111363146  rs2823674  rs12605964  rs115023714  rs72636644  rs56821385  rs12054057  rs35650976  rs9951447  rs12140153  rs9929143  rs56143801  rs855679  rs11138311  rs112661041  rs145990041  rs77931950  rs150832462  rs7488791  rs4516268  rs2311597  rs9974178  rs62370472  rs352126  rs13021948  rs6668050  rs11001477  rs1546721  rs7314285  rs11128901  rs9788721  rs114697636  rs62111724  rs67514550  rs56823429  rs2378253  rs17040284  rs198851  rs62158591  rs62244890  rs79896703  rs77960347  rs55695634  rs10027182 |
| **HDL-Cholesterol** |  |  |  |
| *Causal Inference for Genetically Determined Levels of High-Density Lipoprotein Cholesterol and Risk of Infectious Disease* | Trinder M, Walley KR, Boyd JH, Brunham LR. Causal Inference for Genetically Determined Levels of High-Density Lipoprotein Cholesterol and Risk of Infectious Disease. Arterioscler Thromb Vasc Biol. 2020;40(1):267-278. | 2020 | rs1121980  rs11869286  rs12145743  rs12328675  rs12678919  rs12748152  rs12801636  rs13107325  rs13326165  rs1532085  rs1689800  rs16942887  rs17145738  rs17173637  rs17404153  rs174546  rs17695224  rs1800961  rs181362  rs1883025  rs2293889  rs2602836  rs2606736  rs2923084  rs2925979  rs2954029  rs2972146  rs3136441  rs3764261  rs386000  rs4129767  rs4420638  rs4650994  rs4660293  rs4731702  rs4759375  rs4765127  rs4846914  rs4917014  rs4983559  rs499974  rs581080  rs605066  rs6450176  rs702485  rs7134375  rs7134594  rs7241918  rs7255436  rs731839  rs7337337  rs7941030  rs838880  rs964184  rs970548  rs998584  rs9987289 |
| *Using a two-sample Mendelian randomization design to investigate a possible causal effect of maternal lipid concentrations on offspring birth weight* | Hwang LD, Lawlor DA, Freathy RM, Evans DM, Warrington NM. Using a two-sample Mendelian randomization design to investigate a possible causal effect of maternal lipid concentrations on offspring birth weight. *Int J Epidemiol*. 2019;48(5):1457-1467. | 2019 | rs4660293  rs12133576  rs646776  rs12145743  rs4650994  rs1689797  rs2642438  rs4846914  rs6680658  rs1367117  rs7607980  rs355838  rs1047891  rs1515110  rs2290547  rs2240327  rs13326165  rs6805251  rs687339  rs1482852  rs10019888  rs442177  rs3822072  rs2602836  rs13107325  rs6450176  rs9686661  rs4976033  rs205262  rs998584  rs9491696  rs634869  rs12525163  rs702485  rs17286602  rs10282707  rs4917014  rs17145738  rs3996352  rs17173637  rs4240624  rs4332136  rs12678919  rs894210  rs2293889  rs4871137  rs2980885  rs2954022  rs4075205  rs686030  rs1883025  rs2472509  rs970548  rs7897379  rs2068888  rs2255141  rs2923084  rs2303975  rs326214  rs17788930  rs11246602  rs12226802  rs1535  rs12801636  rs499974  rs10790162  rs7117842  rs11045163  rs3741414  rs2241210  rs653178  rs838876  rs10773105  rs4983559  rs2412710  rs492571  rs1532085  rs261342  rs2652834  rs9989419  rs5880  rs16942887  rs2925979  rs931992  rs4148005  rs4969178  rs4939883  rs11660468  rs952044  rs2278236  rs731839  rs17695224  rs103294  rs1800961  rs4465830  rs181362 |
| *Causal Factors for Knee, Hip, and Hand Osteoarthritis: A Mendelian Randomization Study in the UK Biobank* | Funck-Brentano T, Nethander M, Movérare-Skrtic S, Richette P, Ohlsson C. Causal Factors for Knee, Hip, and Hand Osteoarthritis: A Mendelian Randomization Study in the UK Biobank. *Arthritis Rheumatol*. 2019;71(10):1634-1641. | 2019 | rs12748152  rs12145743  rs4650994  rs1689800  rs2972146  rs13326165  rs2602836  rs1936800  rs605066  rs4142995  rs4917014  rs17173637  rs9987289  rs581080  rs970548  rs29223084  rs12801636  rs499974  rs964184  rs11613352  rs4759375  rs838880  rs4983559  rs1532085  rs2652834  rs3764261  rs16942887  rs2925979  rs11869286  rs4148008  rs7241918  rs7255436  rs737337  rs17695224  rs1800961  rs181362 |
| *LDL Cholesterol* |  |  |  |
| *Genetically-predicted life-long lowering of low-density lipoprotein cholesterol is associated with decreased frailty: A Mendelian randomization study in UK biobank* | Wang Q, Wang Y, Lehto K, Pedersen NL, Williams DM, Hägg S. Genetically-predicted life-long lowering of low-density lipoprotein cholesterol is associated with decreased frailty: A Mendelian randomization study in UK biobank. EBioMedicine. 2019;45:487-494. | 2019 | rs10410  rs10903129  rs10910490  rs11102964  rs11206510  rs11206514  rs11485618  rs11581665  rs11583974  rs11591147  rs12066643  rs12127701  rs12129277  rs12410656  rs12748152  rs1278286  rs1337247  rs13375691  rs1386585  rs1475701  rs17035630  rs17035665  rs17647543  rs1874776  rs207145  rs2131925  rs2247213  rs2479394  rs2479409  rs2587534  rs2642438  rs2642442  rs2647281  rs267733  rs413380  rs413582  rs4847221  rs4927207  rs4970712  rs572512  rs585131  rs585362  rs629301  rs646776  rs650985  rs6662286  rs6689614  rs72703204  rs7512480  rs7544735  rs7552841  rs10195252  rs10208987  rs1025447  rs10490626  rs11096689  rs11563251  rs11679386  rs11685356  rs12471982  rs1250229  rs12691202  rs12720796  rs12720842  rs13027175  rs1367117  rs1534420  rs16831243  rs17398765  rs17508045  rs2030746  rs2194562  rs2710642  rs312049  rs3791981  rs4148177  rs4148218  rs4299376  rs492399  rs4953023  rs4988235  rs515135  rs6413458  rs6544713  rs6547409  rs6725189  rs6729410  rs6739502  rs6754295  rs6756743  rs6759321  rs75279593  rs7567653  rs780093  rs11709504  rs17404153  rs17819328  rs7640978  rs9875338  rs6818397  rs6831256  rs10069744  rs10515198  rs10515214  rs12916  rs16872670  rs3857388  rs4361493  rs4530754  rs4703642  rs4704231  rs4704810  rs6873053  rs6882076  rs7717505  rs7727150  rs10455872  rs117733303  rs12208357  rs1367211  rs1408272  rs1564348  rs16891156  rs1800562  rs2297374  rs2327951  rs2621321  rs3120139  rs3177928  rs3757354  rs3798180  rs3798221  rs389883  rs3918291  rs446218  rs461473  rs6909746  rs6917747  rs6935921  rs7774197  rs9457843  rs12670798  rs2073547  rs217386  rs4719841  rs4722551  rs6461566  rs10102164  rs10102352  rs11136341  rs13277801  rs2081687  rs2737252  rs2980875  rs4360309  rs4592055  rs7832643  rs9987289  rs11244084  rs11795315  rs1883025  rs3780181  rs4489379  rs579459  rs630014  rs7030248  rs8176693  rs8176720  rs2255141  rs2419604  rs10832962  rs10893493  rs10893499  rs10893505  rs11220462  rs11600380  rs12294259  rs174476  rs174546  rs174583  rs180326  rs2075290  rs2845573  rs508487  rs582037  rs10850003  rs11065987  rs11066028  rs1169288  rs17630235  rs2708101  rs3184504  rs657197  rs7953150  rs4942486  rs8017377  rs12448528  rs12931964  rs1864163  rs2000999  rs217181  rs247616  rs3764261  rs7197967  rs8044335  rs8044476  rs8060878  rs9302635  rs1801689  rs2886232  rs314253  rs6504872  rs7206971  rs8070463  rs10401969  rs10402271  rs10403668  rs10419669  rs10422616  rs10460181  rs1048699  rs11668536  rs11669133  rs11881156  rs12150984  rs12721109  rs1529711  rs1531517  rs157580  rs1594895  rs16979372  rs16996148  rs17677316  rs17800760  rs1799898  rs2075650  rs2287019  rs2315025  rs2927477  rs2965101  rs2965157  rs2965174  rs2972564  rs3208856  rs36005514  rs376642  rs3786721  rs3786722  rs379309  rs387976  rs4420638  rs445925  rs4803760  rs4803766  rs492602  rs5158  rs5742911  rs59325138  rs6511720  rs6859  rs688  rs714948  rs7251031  rs7252981  rs7255743  rs73015030  rs73045960  rs77301115  rs8102380  rs8103315  rs8108762  rs892114  rs926054  rs9973305  rs1800961  rs2328223  rs2745865  rs2865507  rs2902940  rs364585  rs4142393  rs4812494  rs6016381  rs6065311  rs6124309  rs7264396  rs742748  rs4253772  rs4253776  rs5763662  rs76733602  rs68160747  rs72768351  rs4703665  rs6877840  rs17244939  rs5908  rs74695562  rs7706933  rs3804231  rs80324692  rs75944831  rs62366598  rs11206479  rs10218716  rs2500340  rs943645  rs12093385  rs12075602  rs17111474  rs2479398  rs2864123  rs2479396  rs17111483  rs17111490  rs12739979  rs11810371  rs77406753  rs2479406  rs28385701  rs74700387  rs28385708  rs11800243  rs12067569  rs10493176  rs17111657  rs960502  rs12138592  rs80355736  rs117175530  rs4724302  rs73107405  rs113496141  rs6956388  rs10234070  rs76236858  rs76331929  rs217437  rs35349497  rs41279627  rs113223818  rs56243746  rs117843715  rs117623941  rs2008036  rs77516609  rs7792931  rs118187778  rs79836087  rs76446485  rs80169634  rs76203970  rs114776298  rs1801695  rs1801700  rs72653053  rs62122522  rs114368165  rs115071638  rs12470778  rs312966  rs113588790  rs72782175  rs1801703  rs1801699  rs79112951  rs17519079  rs1263149  rs34552724  rs78296522  rs74773964  rs633389  rs5110  rs11216164  rs548638  rs11216169  rs543819  rs17174502  rs7943309  rs7106782  rs17678136  rs13345127  rs2072382  rs2421198  rs17242346  rs12981050  rs17242367  rs17248748  rs2569556  rs6413503  rs3826810  rs72658879  rs4804146  rs892113  rs4804149  rs11880059  rs17616661  rs4804150  rs4804576 |
| *Using a two-sample Mendelian randomization design to investigate a possible causal effect of maternal lipid concentrations on offspring birth weight* | Hwang LD, Lawlor DA, Freathy RM, Evans DM, Warrington NM. Using a two-sample Mendelian randomization design to investigate a possible causal effect of maternal lipid concentrations on offspring birth weight. *Int J Epidemiol*. 2019;48(5):1457-1467. | 2019 | rs267733  rs1367117  rs11563251  rs7640978  rs6882076  rs1564348  rs12670798  rs4722551  rs10102164  rs3780181  rs1169288  rs4942486  rs8017377  rs10401969  rs364585  rs2328223  rs5763662  rs10903129  rs646776  rs2642438  rs2587534  rs515135  rs6544713  rs4148218  rs2710642  rs17508045  rs2030746  rs16831243  rs1250229  rs9875338  rs4530754  rs1800562  rs2297374  rs2073547  rs217386  rs2737252  rs7832643  rs1883025  rs8176720  rs579459  rs2255141  rs10832962  rs11220462  rs2000999  rs314253  rs6511720  rs688  rs6859  rs492602  rs7264396  rs6016381  rs6065311  rs1800961  rs1998013  rs4587594  rs6603981  rs1010167  rs903319  rs1260326  rs3817588  rs2287623  rs17345563  rs7703051  rs2294261  rs2247056  rs17789218  rs868943  rs4240624  rs2326077  rs2980885  rs2954022  rs174532  rs1535  rs10790162  rs653178  rs6489818  rs1186380  rs9989419  rs2288002  rs4791641  rs7225700  rs7254892 |
| *Causal Factors for Knee, Hip, and Hand Osteoarthritis: A Mendelian Randomization Study in the UK Biobank* | Funck-Brentano T, Nethander M, Movérare-Skrtic S, Richette P, Ohlsson C. Causal Factors for Knee, Hip, and Hand Osteoarthritis: A Mendelian Randomization Study in the UK Biobank. *Arthritis Rheumatol*. 2019;71(10):1634-1641. | 2019 | rs12027135  rs12748152  rs2479409  rs2131925  rs629301  rs267733  rs2642442  rs514230  rs1367117  rs4299376  rs11563251  rs7640978  rs17404153  rs6818397  rs6882076  rs3757354  rs3177928  rs1564348  rs12670798  rs4722551  rs2072183  rs9987289  rs10102164  rs11136341  rs3780181  rs635634  rs964184  rs1169288  rs4942486  rs8017377  rs3764261  rs1801689  rs10401969  rs364585  rs2328223  rs6029526  rs5763662 |
| *Causal Inference for Genetically Determined Levels of High-Density Lipoprotein Cholesterol and Risk of Infectious Disease* | Trinder M, Walley KR, Boyd JH, Brunham LR. Causal Inference for Genetically Determined Levels of High-Density Lipoprotein Cholesterol and Risk of Infectious Disease. Arterioscler Thromb Vasc Biol. 2020;40(1):267-278. | 2020 | rs1121980  rs11869286  rs12145743  rs12328675  rs12678919  rs12748152  rs12801636  rs13107325  rs13326165  rs1532085  rs1689800  rs16942887  rs17145738  rs17173637  rs17404153  rs174546  rs17695224  rs1800961  rs181362  rs1883025  rs2293889  rs2602836  rs2606736  rs2923084  rs2925979  rs2954029  rs2972146  rs3136441  rs3764261  rs386000  rs4129767  rs4420638  rs4650994  rs4660293  rs4731702  rs4759375  rs4765127  rs4846914  rs4917014  rs4983559  rs499974  rs581080  rs605066  rs6450176  rs702485  rs7134375  rs7134594  rs7241918  rs7255436  rs731839  rs7337337  rs7941030  rs838880  rs964184  rs970548  rs998584  rs9987289 |
| **Triglycerides** |  |  |  |
| *Using a two-sample Mendelian randomization design to investigate a possible causal effect of maternal lipid concentrations on offspring birth weight* | Hwang LD, Lawlor DA, Freathy RM, Evans DM, Warrington NM. Using a two-sample Mendelian randomization design to investigate a possible causal effect of maternal lipid concentrations on offspring birth weight. *Int J Epidemiol*. 2019;48(5):1457-1467. | 2019 | rs4846914  rs10493326  rs4587594  rs1367117  rs1260326  rs3817588  rs7607980  rs687339  rs1515110  rs10513688  rs6831256  rs442177  rs10029254  rs9686661  rs6882076  rs2247056  rs998584  rs634869  rs4722551  rs17145738  rs799160  rs38855  rs9693857  rs4921914  rs12678919  rs894210  rs2980885  rs2954022  rs7033354  rs1883025  rs1781930  rs7897379  rs2068888  rs2255141  rs326214  rs1535  rs10790162  rs603446  rs3741414  rs10861661  rs1341267  rs2412710  rs492571  rs1532085  rs261342  rs2652834  rs1035744  rs3198697  rs749671  rs9930333  rs9989419  rs5880  rs2925979  rs8077889  rs10401969  rs731839  rs1688030  rs7254892  rs4465830  rs3761445 |
| *Causal Factors for Knee, Hip, and Hand Osteoarthritis: A Mendelian Randomization Study in the UK Biobank* | Funck-Brentano T, Nethander M, Movérare-Skrtic S, Richette P, Ohlsson C. Causal Factors for Knee, Hip, and Hand Osteoarthritis: A Mendelian Randomization Study in the UK Biobank. *Arthritis Rheumatol*. 2019;71(10):1634-1641. | **2019** | rs12748152  rs2131925  rs2972146  rs6831256  rs9686661  rs6882076  rs719726  rs4719841  rs38855  rs1495741  rs964184  rs11613352  rs1532085  rs3198697  rs3764261  rs8077889  rs7248104  rs10401969 |
| *Causal Inference for Genetically Determined Levels of High-Density Lipoprotein Cholesterol and Risk of Infectious Disease* | Trinder M, Walley KR, Boyd JH, Brunham LR. Causal Inference for Genetically Determined Levels of High-Density Lipoprotein Cholesterol and Risk of Infectious Disease. Arterioscler Thromb Vasc Biol. 2020;40(1):267-278. | 2020 | rs1121980  rs11869286  rs12145743  rs12328675  rs12678919  rs12748152  rs12801636  rs13107325  rs13326165  rs1532085  rs1689800  rs16942887  rs17145738  rs17173637  rs17404153  rs174546  rs17695224  rs1800961  rs181362  rs1883025  rs2293889  rs2602836  rs2606736  rs2923084  rs2925979  rs2954029  rs2972146  rs3136441  rs3764261  rs386000  rs4129767  rs4420638  rs4650994  rs4660293  rs4731702  rs4759375  rs4765127  rs4846914  rs4917014  rs4983559  rs499974  rs581080  rs605066  rs6450176  rs702485  rs7134375  rs7134594  rs7241918  rs7255436  rs731839  rs7337337  rs7941030  rs838880  rs964184  rs970548  rs998584  rs9987289 |

**S2. ICD-10 and OPCS-4 coding and descriptions**

| **International Classification of Disease, tenth revision (ICD-10)** |
| --- |
| I20X Angina pectoris |
| I21X Acute myocardial infarction  I22X Subsequent myocardial infarction  I23X Current complications following acute myocardial infarction  I24X Other acute ischemic heart diseases  I25.1 Atherosclerotic heart disease  25.2 Old myocardial infarction  I25.5 Ischaemic cardiomyopathy  I25.6 Silent myocardial ischemia  I25.8 Other forms of chronic ischaemic heart disease  I25.9 Chronic ischaemic heart disease, unspecified) |
| I23X Current complications following acute myocardial infarction |
| I24X Other acute ischemic heart diseases |
| I25X I251 Atherosclerotic heart disease  I25.2 Old myocardial infarction  I25.5 Ischaemic cardiomyopath  I25.6 Silent myocardial ischemia  I25.8 Other forms of chronic ischaemic heart disease  I25.9 Chronic ischaemic heart disease, unspecified) |
| K40 Saphenous vein graft replacement of coronary artery  K41 Other autograft replacement of coronary artery  K43 Prosthetic replacement of coronary artery  K46 Other bypass of coronary artery |
| K49 Transluminal balloon angioplasty of coronary artery  K75 Percutaneous transluminal balloon angioplasty and stenting of coronary artery |
| K45 Connection of thoracic artery to coronary artery  K50.1 Percutaneous transluminal laser coronary angioplasty  K50.2 Percutaneoucors transluminal coronary thrombolysis using streptokinase  K50.3 Transluminal atherectomy of coronary artery |

| **Office of Population Censuses and Surveys Classification of Surgical Operations and Procedures, fourth revision (OPCS-4)** |
| --- |
| K40 Saphenous vein graft replacement of coronary artery  K41 Other autograft replacement of coronary artery  K43 Prosthetic replacement of coronary artery  K46 Other bypass of coronary artery |
| K49 Transluminal balloon angioplasty of coronary artery  K75 Percutaneous transluminal balloon angioplasty and stenting of coronary artery |
| K45 Connection of thoracic artery to coronary artery  K50.1 Percutaneous transluminal laser coronary angioplasty  K50.2 Percutaneoucors transluminal coronary thrombolysis using streptokinase  K50.3 Transluminal atherectomy of coronary artery |

**S3. Data-Fields in UK Biobank**

| **Exposures** |
| --- |
| Data-Field 30890 Vitamin D  Data-Field 1558 Alcohol intake frequency  Data-Field 30710 C-reactive protein  Data-Field 30870 Triglycerides  Data-Field 30760 HDL cholesterol  Data-Field 30780 LDL cholesterol |

| **Outcome** |
| --- |
| Data-Field 20002 Non-cancer illness code  Data-Field 20004 Operation code  Data-Field 6150 Vascular/heart problems diagnosed by a doctor  Data-Field 41270 Diagnoses ICD10  Data-Field 41272 Operative procedures OPC4 |

**S4. Supplementary figures 1-6: Flowcharts showing inclusion and exclusion of participants**

Supplementary Figure 1: Flowchart showing inclusion of participants in vitamin D analyses

 
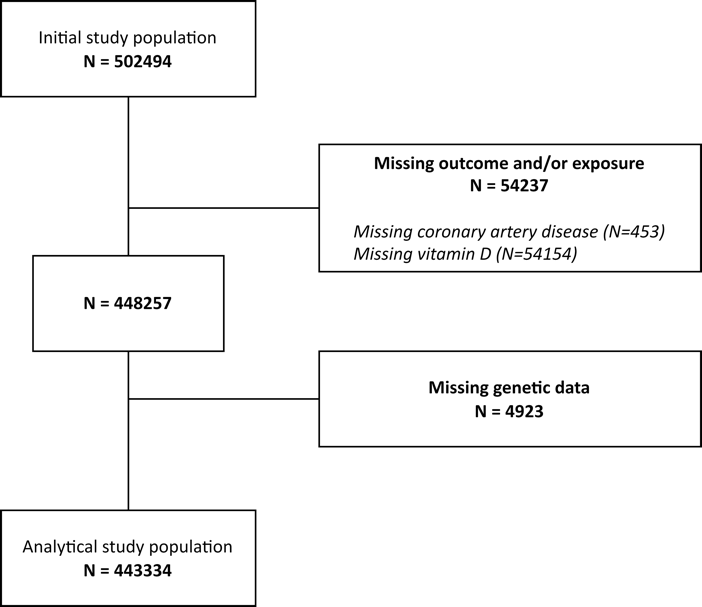


Supplementary Figure 2: Flowchart showing inclusion of participants in CRP analyses

 
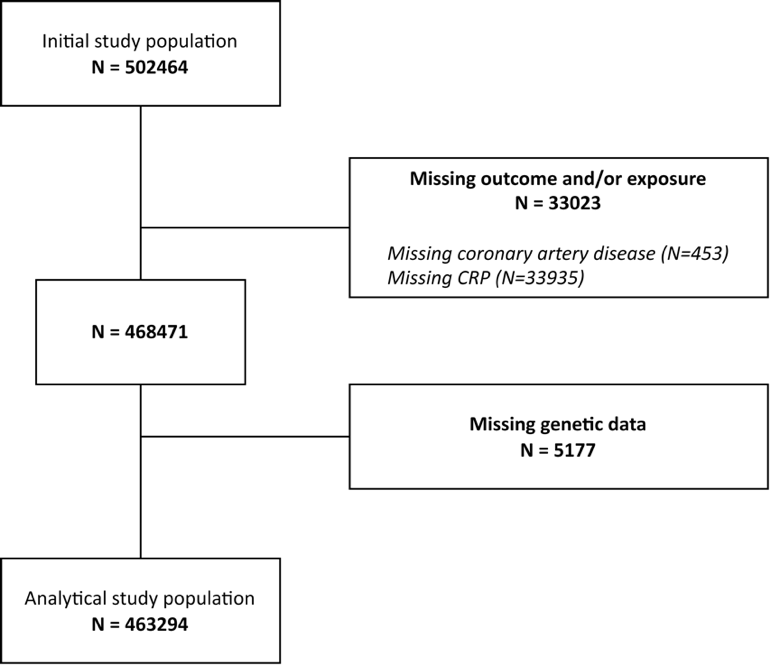


Supplementary Figure 3: Flowchart showing inclusion of participants in alcohol analyses

 
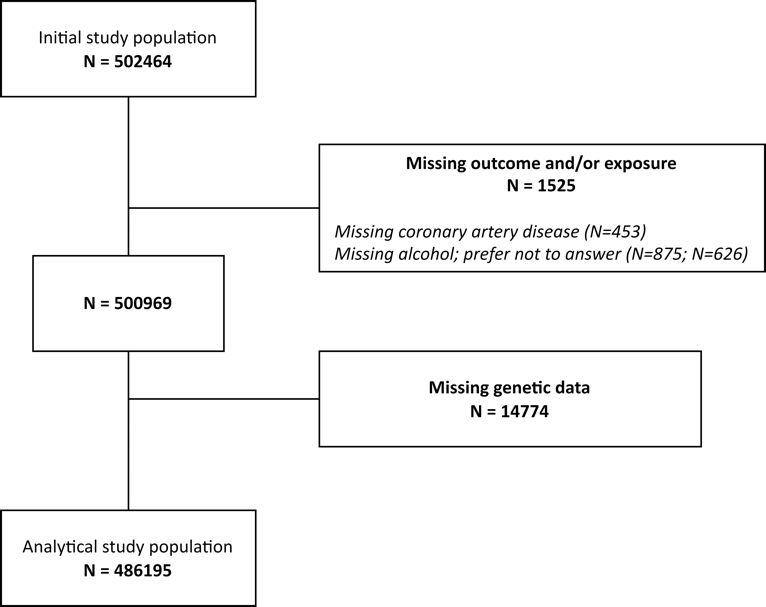


Supplementary Figure 4: Flowchart showing inclusion of participants in triglycerides analyses

 
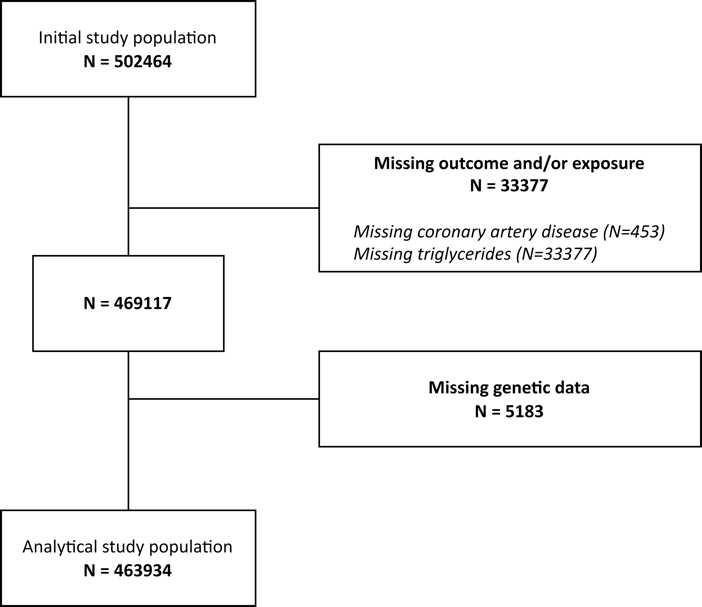


Supplementary Figure 5: Flowchart showing inclusion of participants in HDL-cholesterol analyses

 
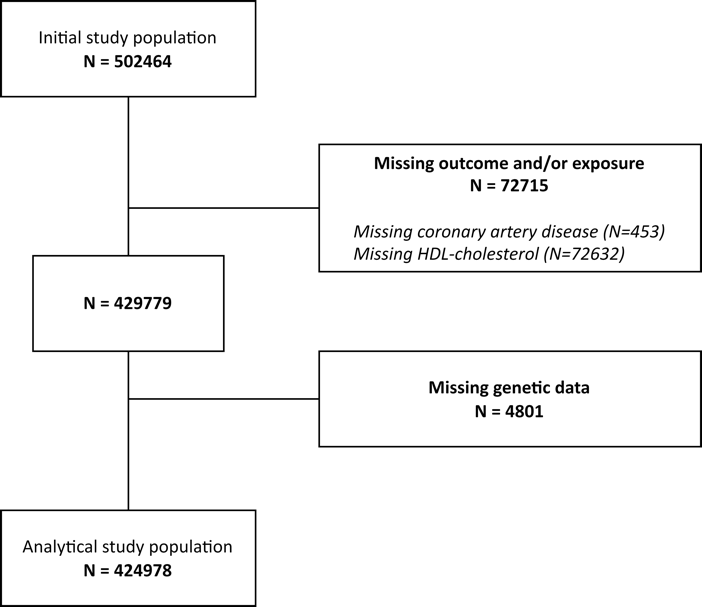


Supplementary Figure 6: Flowchart showing inclusion of participants in LDL-cholesterol analyses

 
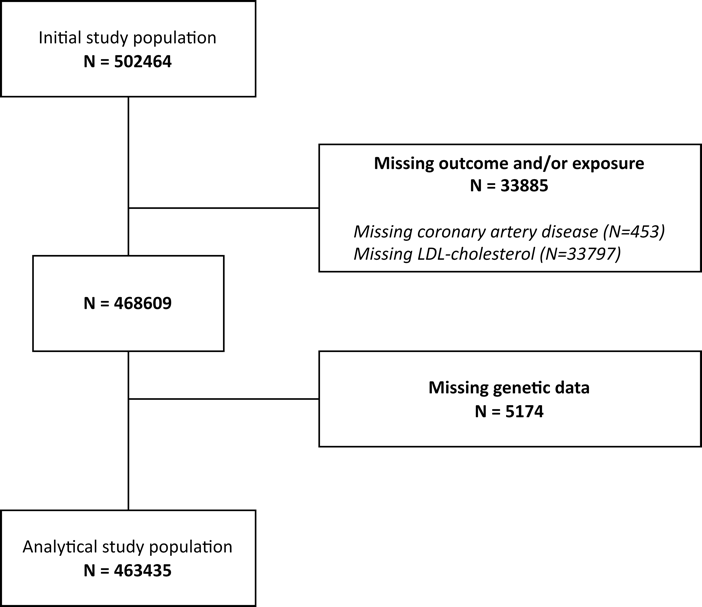


| **Supplementary table 1: Demographic and exposure characteristics of study population** | | | | | | |
| --- | --- | --- | --- | --- | --- | --- |
|  | Study population for analysis with Vitamin D concentration as exposure | Study population for analysis with alcohol consumption as exposure | Study population for analysis with C-reactive protein as exposure | Study population for analysis with triglycerides as exposure | Study population for analysis with HDL-cholesterol as exposure | Study population for analysis with LDL-cholesterol as exposure |
| Sample |  |  |  |  |  |  |
| N | 443,334 | 486,195 | 463,294 | 463,934 | 424,978 | 463,435 |
| Demographics |  |  |  |  |  |  |
| Sex: female | 237,302 (53.5%) | 263,656 (54.2%) | 251,169 (54.2%) | 251,467 (54.2%) | 228,607 (53.8%) | 251,195 (54.2%) |
| Recruitment age in years |  |  |  |  |  |  |
| Median | 58 | 58 | 58 | 58 | 58 | 58 |
| Range | 38 - 73 | 38 - 73 | 38 - 73 | 38 - 73 | 38 – 73 | 38 - 73 |
| Self-reported ethnicity |  |  |  |  |  |  |
| British | 392,142 (88.5%) | 429,905 (88.4%) | 409,326 (88.4%) | 409,879 (88.3%) | 375,464 (88.3%) | 409,448 (88.4%) |
| Irish | 11,705 (2.6%) | 12,701 (2.6%) | 12,113 (2.6%) | 12,126 (2.6%) | 11,090 (2.6%) | 12,110 (2.6%) |
| Any other white background | 14,318 (3.2%) | 15,745 (3.2%) | 14,952 (3.2%) | 14,974 (3.2%) | 13,690 (3.2%) | 14,957 (3.2%) |
| Other | 25,169(5.7%) | 27,844 (5.7%) | 26,906 (5.8%) | 26,955 (5.8%) | 24,734 (5.8%) | 26,920 (5.8%) |
| Outcome |  |  |  |  |  |  |
| Coronary artery disease | 21,496 (4.8%) | 22,964 (4.7%) | 21,817 (4.7%) | 21,859 (4.7%) | 20,062 (4.7%) | 21,838 (4.7%) |
| Exposures |  |  |  |  |  |  |
| Continuous exposures: vitamin D, CRP, triglycerides, HDL-cholesterol, LDL-cholesterol |  |  |  |  |  |  |
| Mean [SD] | 48.64 nmol/L [21.1] | N.A. | 2.60 mg/L [4.4] | 1.75 mmol/L[1.03] | 1.45 [0.38] mmol/L | 3.56 [0.87] mmol/L |
| Categorical exposures: alcohol consumption |  |  |  |  |  |  |
| ‘Never’ | N.A. | 39,163 (8.1%) | N.A. | N.A. | N.A. | N.A. |
| ‘Special occasions only’ | N.A. | 55,958 (11.5%) | N.A. | N.A. | N.A. | N.A. |
| ‘One to three times a month’ | N.A. | 54,141 (11.1%) | N.A. | N.A. | N.A. | N.A. |
| ‘Once or twice a week’ | N.A. | 125,500 (25.8%) | N.A. | N.A. | N.A. | N.A. |
| ‘Three to four times a week’ | N.A. | 112,390 (23.1%) | N.A. | N.A. | N.A. | N.A. |
| ‘Daily or almost daily’ | N.A. | 99,043 (20.4%) | N.A. | N.A. | N.A. | N.A. |

**S5. Supplementary table 1: Demographic and exposure characteristics of study population**

**S6. Toy example**

The instrumental inequalities are defined as:

$$\max_{x} \sum_{y} \max_{z} P\left( x,y \mid z \right)\leq1$$

where *z* is the proposed instrument, *x* is the exposure and *y* is the outcome. We apply the instrumental inequalities to two settings, referred to as A and B. In A, *z* is a valid instrument variable for the effect of *x* on *y*. In B, the instrumental conditions are violated by a direct effect of *z* on *y*.

**A B**


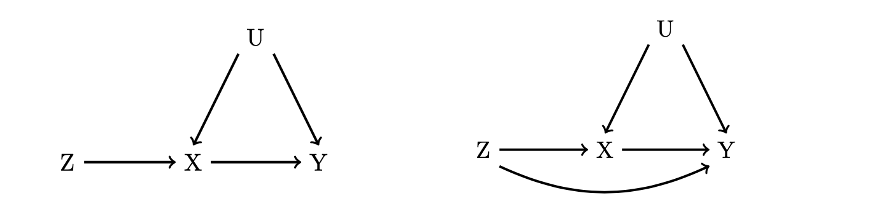


The R code for this toy example can be found in section 10 of this supplement. In both settings A and B, the sample size is 100 000, variable *z* can take the value of 0, 1 or 2 and *x* is a binary variable.

| **A** | | | |
| --- | --- | --- | --- |
|  | Z | X | Y |
| 1 | 0 | 1 | 1 |
| 2 | 1 | 0 | 0 |
| 3 | 2 | 1 | 0 |
| … | … | … | … |
| 99 998 | 0 | 1 | 1 |
| 99 999 | 1 | 1 | 1 |
| 100 000 | 1 | 0 | 0 |

| **B** | | | |
| --- | --- | --- | --- |
|  | Z | X | Y |
| 1 | 0 | 1 | 1 |
| 2 | 1 | 0 | 1 |
| 3 | 2 | 1 | 1 |
| … | … | … | … |
| 99 998 | 0 | 1 | 1 |
| 99 999 | 1 | 1 | 0 |
| 100 000 | 1 | 0 | 1 |

First, we compute the proportions of each possible combination of x and y given z.

| **A** | | | |  |  | | | | |  | |  | | | | |
| --- | --- | --- | --- | --- | --- | --- | --- | --- | --- | --- | --- | --- | --- | --- | --- | --- |
| **Z=0** | | | |  | **Z=1** | | | | |  | | **Z=2** | | | | |
|  |  | **Y** | |  |  |  | **Y** | | |  | |  |  | **Y** | |  |
|  |  | **0** | **1** |  |  |  | **0** | **1** |  | |  | |  | **0** | **1** |  |
| **X** | **0** | 0.640 | 0.159 |  | **X** | **0** | 0.536 | 0.132 |  | | **X** | | **0** | 0.400 | 0.099 |  |
|  | **1** | 0.133 | 0.068 |  |  | **1** | 0.223 | 0.108 |  | |  |  | **1** | 0.336 | 0.166 |  |

| **B** | | | |  |  | | | | |  | |  | | | | |
| --- | --- | --- | --- | --- | --- | --- | --- | --- | --- | --- | --- | --- | --- | --- | --- | --- |
| **Z=0** | | | |  | **Z=1** | | | | |  | | **Z=2** | | | | |
|  |  | **Y** | |  |  |  | **Y** | | |  | |  |  | **Y** | |  |
|  |  | **0** | **1** |  |  |  | **0** | **1** |  | |  | |  | **0** | **1** |  |
| **X** | **0** | 0.640 | 0.159 |  | **X** | **0** | 0.297 | 0.371 |  | | **X** | | **0** | 0.067 | 0.431 |  |
|  | **1** | 0.133 | 0.068 |  |  | **1** | 0.092 | 0.239 |  | |  |  | **1** | 0.038 | 0.464 |  |

For each unique combination of x and y, we take the maximum value of the proportions.

| **A** | | |
| --- | --- | --- |
| **X** | **Y** | $\max_{Z} P\left( x, y \right\vert z)$ |
| 0 | 0 | 0.640 |
| 0 | 1 | 0.159 |
| 1 | 0 | 0.336 |
| 1 | 1 | 0.166 |

| **B** | | |
| --- | --- | --- |
| **X** | **Y** | $\max_{Z} P\left( x, y \right\vert z)$ |
| 0 | 0 | 0.640 |
| 0 | 1 | 0.431 |
| 1 | 0 | 0.133 |
| 1 | 1 | 0.464 |

We sum these maximum values over y.

| **A** | |
| --- | --- |
| **X** | $\sum_{y} \max_{z} P\left( x, y \right\vert z)$ |
| 0 | 0.799 |
| 1 | 0.502 |

| **B** | |
| --- | --- |
| **X** | $\sum_{y} \max_{z} P\left( x, y \right\vert z)$ |
| 0 | 1.071 |
| 1 | 0.597 |

Because all sums have to be less than or equal to one, we take the maximum value over all sums. In A and B these are, respectively, $\max_{x} \sum_{y} \max_{z} P\left( x,y \mid z \right)=0.799$and $\max_{x} \sum_{y} \max_{z} P\left( x,y \mid z \right)=1.071.$

The maximum is compared to 1 to test if the instrumental inequalities hold or not. If the sums of the maximum proportions are equal or less than one the instrumental inequalities hold. If the sum of the observed probabilities is greater than one, it implies that at least one of the instrumental conditions is violated within the dataset.

**S7. Details of inverse probability weighting procedure**

For each proposed instrument Z, unstabilized inverse probability weights (W^A^) were estimated as follows, using software developed by Diemer et al 2020:

$$\boldsymbol{W}^{\boldsymbol{A}}\boldsymbol{=}\frac{\boldsymbol{1}}{\boldsymbol{P(Z|P}\boldsymbol{C}_{\boldsymbol{1}}\boldsymbol{,P}\boldsymbol{C}_{\boldsymbol{2}}\boldsymbol{,P}\boldsymbol{C}_{\boldsymbol{3}}\boldsymbol{,P}\boldsymbol{C}_{\boldsymbol{4}}\boldsymbol{,P}\boldsymbol{C}_{\boldsymbol{5}}\boldsymbol{,P}\boldsymbol{C}_{\boldsymbol{6}}\boldsymbol{,P}\boldsymbol{C}_{\boldsymbol{7}}\boldsymbol{,P}\boldsymbol{C}_{\boldsymbol{8}}\boldsymbol{,P}\boldsymbol{C}_{\boldsymbol{9}}\boldsymbol{,P}\boldsymbol{C}_{\boldsymbol{10}}\boldsymbol{)}}$$

To estimate W^A^, we fitted multinomial logistic regression models predicting Z, assuming principal components (PC) contributed additively and linearly on the logit scale. Individual values of W^A^ = 1/P(Z |PC_1_, PC_2_, PC_3_, PC_4_, PC_5_, PC_6_, PC_7_, PC_8_, PC_9_, PC_10_) were then calculated for each individual using values back-transformed to probabilities.

**S8. Supplementary tables 2-7: Results of the instrumental inequalities for each SNP proposed as an instrument**

| **Supplementary Table 2: Results of Instrumental Inequalities applied to MR models for the effect of vitamin D concentration on coronary artery disease** | | |  |
| --- | --- | --- | --- |
| **Proposed instrument** | **Value of instrumental inequalities** | **Do instrumental inequalities hold?** | |
| rs12785878 | 0.200 | Yes | |
| rs6013897 | 0.121 | Yes | |
| rs10741657 | 0.146 | Yes | |
| rs12794714 | 0.132 | Yes | |
| rs116970203 | 0.358 | Yes | |
| rs3755967 | 0.135 | Yes | |
| rs17216707 | 0.109 | Yes | |
| rs10745742 | 0.103 | Yes | |
| rs8018720 | 0.120 | Yes | |
| rs117913124 | 0.351 | Yes | |
| rs2282679 | 0.135 | Yes | |
| rs7944926 | 0.200 | Yes | |
| rs11234027 | 0.174 | Yes | |
| rs4588 | 0.135 | Yes | |
| rs4423214 | 0.198 | Yes | |
| Allele score | 0.553 | Yes | |

| **Supplementary Table 3: Results of instrumental inequalities for MR models for the effect of alcohol consumption on coronary artery disease** | | |
| --- | --- | --- |
| **Proposed instrument** | **Value of instrumental inequalities** | **Do instrumental inequalities hold?** |
| rs11039429 | 0.259 | Yes |
| rs11787216 | 0.267 | Yes |
| rs11940694 | 0.260 | Yes |
| rs13102973 | 0.262 | Yes |
| rs13231886 | 0.261 | Yes |
| rs13390019 | 0.261 | Yes |
| rs17097556 | 0.259 | Yes |
| rs17690703 | 0.270 | Yes |
| rs1788030 | 0.261 | Yes |
| rs1893659 | 0.259 | Yes |
| rs2159935 | 0.259 | Yes |
| rs363096 | 0.260 | Yes |
| rs4726481 | 0.260 | Yes |
| rs540606 | 0.263 | Yes |
| rs571312 | 0.259 | Yes |
| rs58905411 | 0.262 | Yes |
| rs6016781 | 0.261 | Yes |
| rs650558 | 0.262 | Yes |
| rs7428430 | 0.262 | Yes |
| rs838145 | 0.262 | Yes |
| rs9372625 | 0.259 | Yes |
| rs9842406 | 0.261 | Yes |
| rs9923768 | 0.259 | Yes |
| rs145452708 | 0.265 | Yes |
| rs193099203 | 0.265 | Yes |
| rs29001570 | 0.337 | Yes |
| rs9841829 | 0.261 | Yes |
| rs1229984 | 0.315 | Yes |
| Allele Score | 1.111 | *No* |

| **Supplementary Table 4: Results of instrumental inequalities for MR models for the effect of C-Reactive Protein on coronary artery disease** | | |
| --- | --- | --- |
| **Proposed instrument** | **Value of instrumental inequalities** | **Do instrumental inequalities hold?** |
| rs10027182 | 0.106 | Yes |
| rs10083137 | 0.125 | Yes |
| rs10095930 | 0.106 | Yes |
| rs10106298 | 0.105 | Yes |
| rs10203386 | 0.104 | Yes |
| rs1037170 | 0.106 | Yes |
| rs1045075 | 0.102 | Yes |
| rs10453441 | 0.102 | Yes |
| rs10490871 | 0.104 | Yes |
| rs1052067 | 0.105 | Yes |
| rs1056441 | 0.107 | Yes |
| rs10752652 | 0.103 | Yes |
| rs10755296 | 0.105 | Yes |
| rs10760691 | 0.105 | Yes |
| rs10779035 | 0.103 | Yes |
| rs10794644 | 0.105 | Yes |
| rs10802496 | 0.108 | Yes |
| rs10806385 | 0.103 | Yes |
| rs10810455 | 0.103 | Yes |
| rs10831676 | 0.103 | Yes |
| rs10848035 | 0.104 | Yes |
| rs10849773 | 0.188 | Yes |
| rs10864086 | 0.106 | Yes |
| rs10924372 | 0.104 | Yes |
| rs10957159 | 0.107 | Yes |
| rs11000760 | 0.106 | Yes |
| rs11001477 | 0.107 | Yes |
| rs11012732 | 0.103 | Yes |
| rs11047139 | 0.106 | Yes |
| rs11057214 | 0.120 | Yes |
| rs11065050 | 0.124 | Yes |
| rs11078597 | 0.104 | Yes |
| rs11083166 | 0.103 | Yes |
| rs111237020 | 0.118 | Yes |
| rs11128901 | 0.104 | Yes |
| rs111363146 | 0.107 | Yes |
| rs11138311 | 0.112 | Yes |
| rs111435864 | 0.112 | Yes |
| rs111570031 | 0.127 | Yes |
| rs11158975 | 0.105 | Yes |
| rs11210869 | 0.103 | Yes |
| rs11222703 | 0.102 | Yes |
| rs11227395 | 0.105 | Yes |
| rs112283362 | 0.114 | Yes |
| rs11229252 | 0.106 | Yes |
| rs11231161 | 0.103 | Yes |
| rs11246574 | 0.107 | Yes |
| rs112661041 | 0.112 | Yes |
| rs112689575 | 0.124 | Yes |
| rs112750178 | 0.105 | Yes |
| rs114451718 | 0.173 | Yes |
| rs114574084 | 0.134 | Yes |
| rs114697636 | 0.124 | Yes |
| rs114733917 | 0.136 | Yes |
| rs114947103 | 0.106 | Yes |
| rs115023714 | 0.136 | Yes |
| rs115093996 | 0.159 | Yes |
| rs115178836 | 0.169 | Yes |
| rs115273594 | 0.181 | Yes |
| rs115462819 | 0.126 | Yes |
| rs11577023 | 0.104 | Yes |
| rs116073407 | 0.147 | Yes |
| rs116424768 | 0.157 | Yes |
| rs11648192 | 0.103 | Yes |
| rs11648261 | 0.106 | Yes |
| rs11652684 | 0.105 | Yes |
| rs11666245 | 0.117 | Yes |
| rs11667234 | 0.113 | Yes |
| rs11672020 | 0.103 | Yes |
| rs116804861 | 0.104 | Yes |
| rs11681145 | 0.104 | Yes |
| rs11682186 | 0.105 | Yes |
| rs11693537 | 0.105 | Yes |
| rs11693697 | 0.105 | Yes |
| rs116971887 | 0.146 | Yes |
| rs11708067 | 0.103 | Yes |
| rs11711864 | 0.103 | Yes |
| rs117211511 | 0.117 | Yes |
| rs117264457 | 0.149 | Yes |
| rs117368206 | 0.167 | Yes |
| rs117548270 | 0.174 | Yes |
| rs11767338 | 0.106 | Yes |
| rs117695016 | 0.117 | Yes |
| rs11772703 | 0.102 | Yes |
| rs117783785 | 0.145 | Yes |
| rs117803005 | 0.134 | Yes |
| rs117849038 | 0.156 | Yes |
| rs11928797 | 0.106 | Yes |
| rs12054057 | 0.106 | Yes |
| rs12055445 | 0.103 | Yes |
| rs12077265 | 0.119 | Yes |
| rs12132412 | 0.103 | Yes |
| rs12138486 | 0.105 | Yes |
| rs12140153 | 0.108 | Yes |
| rs12141189 | 0.106 | Yes |
| rs12211604 | 0.108 | Yes |
| rs12231235 | 0.106 | Yes |
| rs12280456 | 0.117 | Yes |
| rs12357890 | 0.103 | Yes |
| rs123698 | 0.103 | Yes |
| rs12425953 | 0.113 | Yes |
| rs12426126 | 0.113 | Yes |
| rs12443906 | 0.104 | Yes |
| rs12448756 | 0.108 | Yes |
| rs12466485 | 0.106 | Yes |
| rs12516176 | 0.106 | Yes |
| rs12517168 | 0.104 | Yes |
| rs12601665 | 0.103 | Yes |
| rs12605964 | 0.102 | Yes |
| rs12620844 | 0.103 | Yes |
| rs12679106 | 0.104 | Yes |
| rs12712928 | 0.107 | Yes |
| rs12714415 | 0.108 | Yes |
| rs12716871 | 0.102 | Yes |
| rs12735458 | 0.137 | Yes |
| rs12828693 | 0.108 | Yes |
| rs1290898 | 0.113 | Yes |
| rs1292067 | 0.105 | Yes |
| rs12933292 | 0.104 | Yes |
| rs12966114 | 0.102 | Yes |
| rs12992747 | 0.108 | Yes |
| rs12992995 | 0.104 | Yes |
| rs13021948 | 0.145 | Yes |
| rs13058527 | 0.106 | Yes |
| rs13066686 | 0.103 | Yes |
| rs13081778 | 0.105 | Yes |
| rs13106834 | 0.103 | Yes |
| rs13108218 | 0.104 | Yes |
| rs13227497 | 0.108 | Yes |
| rs1338721 | 0.104 | Yes |
| rs13416992 | 0.104 | Yes |
| rs1353792 | 0.104 | Yes |
| rs139136389 | 0.184 | Yes |
| rs139460294 | 0.142 | Yes |
| rs139952834 | 0.176 | Yes |
| rs141094656 | 0.143 | Yes |
| rs1412444 | 0.108 | Yes |
| rs141651106 | 0.125 | Yes |
| rs141737681 | 0.222 | Yes |
| rs142603322 | 0.141 | Yes |
| rs143273561 | 0.150 | Yes |
| rs1441043 | 0.106 | Yes |
| rs1441169 | 0.105 | Yes |
| rs144314390 | 0.207 | Yes |
| rs144398877 | 0.133 | Yes |
| rs144970957 | 0.156 | Yes |
| rs145987962 | 0.116 | Yes |
| rs145990041 | 0.215 | Yes |
| rs146342974 | 0.143 | Yes |
| rs146396094 | 0.158 | Yes |
| rs1476698 | 0.104 | Yes |
| rs148059420 | 0.139 | Yes |
| rs148104667 | 0.163 | Yes |
| rs148303016 | 0.135 | Yes |
| rs148770227 | 0.131 | Yes |
| rs148933445 | 0.145 | Yes |
| rs1490384 | 0.107 | Yes |
| rs1492914 | 0.104 | Yes |
| rs1495017 | 0.108 | Yes |
| rs149523036 | 0.152 | Yes |
| rs1495745 | 0.102 | Yes |
| rs149647262 | 0.110 | Yes |
| rs150365073 | 0.170 | Yes |
| rs150377363 | 0.149 | Yes |
| rs150543164 | 0.117 | Yes |
| rs150832462 | 0.131 | Yes |
| rs150844304 | 0.142 | Yes |
| rs150852956 | 0.131 | Yes |
| rs151132887 | 0.149 | Yes |
| rs1545536 | 0.106 | Yes |
| rs1546721 | 0.102 | Yes |
| rs1570868 | 0.107 | Yes |
| rs157512 | 0.106 | Yes |
| rs1635852 | 0.103 | Yes |
| rs16835819 | 0.158 | Yes |
| rs16842320 | 0.132 | Yes |
| rs1693456 | 0.103 | Yes |
| rs17040284 | 0.104 | Yes |
| rs17105232 | 0.104 | Yes |
| rs17138476 | 0.107 | Yes |
| rs17308476 | 0.110 | Yes |
| rs1736057 | 0.106 | Yes |
| rs17366139 | 0.115 | Yes |
| rs17417252 | 0.104 | Yes |
| rs174373 | 0.106 | Yes |
| rs17616063 | 0.149 | Yes |
| rs17626434 | 0.102 | Yes |
| rs17854914 | 0.107 | Yes |
| rs178835 | 0.103 | Yes |
| rs1800693 | 0.103 | Yes |
| rs1800919 | 0.108 | Yes |
| rs1800973 | 0.113 | Yes |
| rs1801272 | 0.120 | Yes |
| rs180686303 | 0.118 | Yes |
| rs182638776 | 0.110 | Yes |
| rs183553104 | 0.163 | Yes |
| rs184334219 | 0.176 | Yes |
| rs185575165 | 0.107 | Yes |
| rs1880241 | 0.107 | Yes |
| rs1886008 | 0.115 | Yes |
| rs1905505 | 0.105 | Yes |
| rs190712692 | 0.113 | Yes |
| rs1933736 | 0.103 | Yes |
| rs1968109 | 0.105 | Yes |
| rs1969026 | 0.125 | Yes |
| rs1975190 | 0.105 | Yes |
| rs1985157 | 0.105 | Yes |
| rs198851 | 0.105 | Yes |
| rs2011689 | 0.103 | Yes |
| rs2030291 | 0.103 | Yes |
| rs204914 | 0.122 | Yes |
| rs2054434 | 0.106 | Yes |
| rs2075803 | 0.103 | Yes |
| rs209474 | 0.103 | Yes |
| rs2107135 | 0.104 | Yes |
| rs2110944 | 0.103 | Yes |
| rs2154384 | 0.146 | Yes |
| rs2161037 | 0.104 | Yes |
| rs2161374 | 0.103 | Yes |
| rs2201637 | 0.108 | Yes |
| rs2207132 | 0.128 | Yes |
| rs2233278 | 0.111 | Yes |
| rs2239222 | 0.108 | Yes |
| rs2239704 | 0.104 | Yes |
| rs2250010 | 0.110 | Yes |
| rs2256027 | 0.120 | Yes |
| rs2265189 | 0.102 | Yes |
| rs2269434 | 0.107 | Yes |
| rs2269841 | 0.105 | Yes |
| rs2301275 | 0.103 | Yes |
| rs2302758 | 0.114 | Yes |
| rs2311597 | 0.103 | Yes |
| rs2378253 | 0.103 | Yes |
| rs2393794 | 0.110 | Yes |
| rs2432195 | 0.109 | Yes |
| rs245768 | 0.105 | Yes |
| rs2472682 | 0.102 | Yes |
| rs2494097 | 0.107 | Yes |
| rs2542170 | 0.106 | Yes |
| rs2627646 | 0.113 | Yes |
| rs263013 | 0.104 | Yes |
| rs2638315 | 0.108 | Yes |
| rs2668244 | 0.107 | Yes |
| rs2681780 | 0.106 | Yes |
| rs2700938 | 0.106 | Yes |
| rs2721953 | 0.105 | Yes |
| rs2740479 | 0.106 | Yes |
| rs2808474 | 0.105 | Yes |
| rs2823674 | 0.103 | Yes |
| rs28361325 | 0.103 | Yes |
| rs2836882 | 0.108 | Yes |
| rs28399607 | 0.120 | Yes |
| rs28601761 | 0.104 | Yes |
| rs288183 | 0.104 | Yes |
| rs2882485 | 0.104 | Yes |
| rs28929474 | 0.142 | Yes |
| rs2927447 | 0.111 | Yes |
| rs2957664 | 0.103 | Yes |
| rs2961111 | 0.104 | Yes |
| rs2965164 | 0.106 | Yes |
| rs2970901 | 0.103 | Yes |
| rs2980512 | 0.102 | Yes |
| rs3014201 | 0.104 | Yes |
| rs301804 | 0.104 | Yes |
| rs3027003 | 0.139 | Yes |
| rs3095122 | 0.102 | Yes |
| rs3125326 | 0.104 | Yes |
| rs3134133 | 0.105 | Yes |
| rs33951980 | 0.120 | Yes |
| rs340005 | 0.106 | Yes |
| rs34061534 | 0.129 | Yes |
| rs34284056 | 0.105 | Yes |
| rs34415150 | 0.111 | Yes |
| rs34761529 | 0.105 | Yes |
| rs34874378 | 0.133 | Yes |
| rs352126 | 0.103 | Yes |
| rs35582280 | 0.103 | Yes |
| rs35650976 | 0.106 | Yes |
| rs35764600 | 0.102 | Yes |
| rs35860194 | 0.159 | Yes |
| rs35881303 | 0.104 | Yes |
| rs36049560 | 0.106 | Yes |
| rs3733892 | 0.104 | Yes |
| rs3736164 | 0.104 | Yes |
| rs3746778 | 0.103 | Yes |
| rs3750310 | 0.103 | Yes |
| rs3751143 | 0.105 | Yes |
| rs3768321 | 0.109 | Yes |
| rs3774063 | 0.109 | Yes |
| rs3785568 | 0.105 | Yes |
| rs3789988 | 0.118 | Yes |
| rs3794204 | 0.111 | Yes |
| rs380825 | 0.102 | Yes |
| rs3808348 | 0.110 | Yes |
| rs3826559 | 0.102 | Yes |
| rs385417 | 0.106 | Yes |
| rs3856270 | 0.103 | Yes |
| rs3865444 | 0.105 | Yes |
| rs4018180 | 0.117 | Yes |
| rs4084164 | 0.103 | Yes |
| rs41290108 | 0.121 | Yes |
| rs4148155 | 0.110 | Yes |
| rs41523449 | 0.108 | Yes |
| rs420957 | 0.104 | Yes |
| rs4239504 | 0.104 | Yes |
| rs424539 | 0.104 | Yes |
| rs4255379 | 0.113 | Yes |
| rs4266763 | 0.106 | Yes |
| rs429358 | 0.227 | Yes |
| rs4411129 | 0.106 | Yes |
| rs4431051 | 0.104 | Yes |
| rs4499304 | 0.102 | Yes |
| rs4506495 | 0.106 | Yes |
| rs4516268 | 0.104 | Yes |
| rs4535048 | 0.107 | Yes |
| rs4641306 | 0.114 | Yes |
| rs4658403 | 0.108 | Yes |
| rs469882 | 0.111 | Yes |
| rs4704780 | 0.114 | Yes |
| rs4714508 | 0.104 | Yes |
| rs4755720 | 0.104 | Yes |
| rs4764939 | 0.106 | Yes |
| rs4766960 | 0.106 | Yes |
| rs4767938 | 0.104 | Yes |
| rs4782568 | 0.102 | Yes |
| rs4788867 | 0.108 | Yes |
| rs480958 | 0.104 | Yes |
| rs4849147 | 0.104 | Yes |
| rs4865540 | 0.107 | Yes |
| rs4871582 | 0.104 | Yes |
| rs4871827 | 0.103 | Yes |
| rs4876992 | 0.104 | Yes |
| rs4915287 | 0.109 | Yes |
| rs4939034 | 0.107 | Yes |
| rs543874 | 0.106 | Yes |
| rs55665939 | 0.105 | Yes |
| rs55695634 | 0.104 | Yes |
| rs55709272 | 0.112 | Yes |
| rs55824262 | 0.104 | Yes |
| rs55855238 | 0.108 | Yes |
| rs55981844 | 0.105 | Yes |
| rs56015600 | 0.107 | Yes |
| rs56094641 | 0.106 | Yes |
| rs56143801 | 0.104 | Yes |
| rs56189574 | 0.104 | Yes |
| rs56821385 | 0.158 | Yes |
| rs56823429 | 0.103 | Yes |
| rs56960368 | 0.106 | Yes |
| rs57550938 | 0.106 | Yes |
| rs58287327 | 0.103 | Yes |
| rs58542926 | 0.121 | Yes |
| rs58770366 | 0.105 | Yes |
| rs59059615 | 0.107 | Yes |
| rs59316512 | 0.157 | Yes |
| rs59737437 | 0.107 | Yes |
| rs601338 | 0.106 | Yes |
| rs6020459 | 0.103 | Yes |
| rs6072279 | 0.103 | Yes |
| rs6073958 | 0.112 | Yes |
| rs6090103 | 0.111 | Yes |
| rs6138537 | 0.102 | Yes |
| rs613872 | 0.106 | Yes |
| rs61542988 | 0.111 | Yes |
| rs61741874 | 0.117 | Yes |
| rs61812598 | 0.123 | Yes |
| rs61821567 | 0.132 | Yes |
| rs62011286 | 0.106 | Yes |
| rs62092069 | 0.103 | Yes |
| rs62106258 | 0.120 | Yes |
| rs62111724 | 0.102 | Yes |
| rs62118504 | 0.106 | Yes |
| rs62121122 | 0.104 | Yes |
| rs62129471 | 0.105 | Yes |
| rs62158591 | 0.110 | Yes |
| rs62204977 | 0.126 | Yes |
| rs62244890 | 0.103 | Yes |
| rs62282070 | 0.112 | Yes |
| rs623011 | 0.105 | Yes |
| rs62370472 | 0.106 | Yes |
| rs62389532 | 0.118 | Yes |
| rs62451586 | 0.110 | Yes |
| rs62491814 | 0.108 | Yes |
| rs62513191 | 0.118 | Yes |
| rs62618693 | 0.122 | Yes |
| rs6265 | 0.111 | Yes |
| rs6433282 | 0.104 | Yes |
| rs6443429 | 0.103 | Yes |
| rs6445393 | 0.108 | Yes |
| rs6447335 | 0.103 | Yes |
| rs645692 | 0.104 | Yes |
| rs6486122 | 0.109 | Yes |
| rs6501199 | 0.110 | Yes |
| rs6501207 | 0.109 | Yes |
| rs6509155 | 0.105 | Yes |
| rs6509222 | 0.104 | Yes |
| rs6519133 | 0.112 | Yes |
| rs653170 | 0.103 | Yes |
| rs654912 | 0.106 | Yes |
| rs6591188 | 0.104 | Yes |
| rs6595549 | 0.108 | Yes |
| rs6595968 | 0.104 | Yes |
| rs663015 | 0.105 | Yes |
| rs6668050 | 0.129 | Yes |
| rs6698653 | 0.116 | Yes |
| rs6705820 | 0.102 | Yes |
| rs674833 | 0.107 | Yes |
| rs67514550 | 0.114 | Yes |
| rs6786055 | 0.106 | Yes |
| rs6792725 | 0.104 | Yes |
| rs68100606 | 0.105 | Yes |
| rs6816467 | 0.127 | Yes |
| rs684016 | 0.102 | Yes |
| rs6840517 | 0.102 | Yes |
| rs6845703 | 0.103 | Yes |
| rs6905544 | 0.105 | Yes |
| rs6920220 | 0.108 | Yes |
| rs6961634 | 0.109 | Yes |
| rs6962836 | 0.107 | Yes |
| rs6984551 | 0.104 | Yes |
| rs7008413 | 0.115 | Yes |
| rs7012637 | 0.108 | Yes |
| rs704017 | 0.103 | Yes |
| rs7084062 | 0.103 | Yes |
| rs7102088 | 0.104 | Yes |
| rs71322200 | 0.112 | Yes |
| rs71414197 | 0.109 | Yes |
| rs71658797 | 0.108 | Yes |
| rs7171864 | 0.103 | Yes |
| rs7189954 | 0.103 | Yes |
| rs72636644 | 0.110 | Yes |
| rs72654472 | 0.135 | Yes |
| rs72660319 | 0.113 | Yes |
| rs72694393 | 0.102 | Yes |
| rs72732974 | 0.113 | Yes |
| rs72743115 | 0.120 | Yes |
| rs7280982 | 0.106 | Yes |
| rs72837690 | 0.103 | Yes |
| rs728538 | 0.111 | Yes |
| rs72959041 | 0.111 | Yes |
| rs7303035 | 0.103 | Yes |
| rs73137144 | 0.104 | Yes |
| rs7314285 | 0.114 | Yes |
| rs7319102 | 0.103 | Yes |
| rs73201521 | 0.117 | Yes |
| rs73577882 | 0.115 | Yes |
| rs74085345 | 0.131 | Yes |
| rs7430523 | 0.111 | Yes |
| rs74354286 | 0.106 | Yes |
| rs7442885 | 0.102 | Yes |
| rs7488791 | 0.103 | Yes |
| rs74949966 | 0.110 | Yes |
| rs7502409 | 0.103 | Yes |
| rs75064168 | 0.108 | Yes |
| rs7528419 | 0.106 | Yes |
| rs7537072 | 0.103 | Yes |
| rs75460349 | 0.147 | Yes |
| rs75497300 | 0.170 | Yes |
| rs7549881 | 0.103 | Yes |
| rs7551731 | 0.157 | Yes |
| rs75898076 | 0.105 | Yes |
| rs75995782 | 0.127 | Yes |
| rs76102184 | 0.126 | Yes |
| rs76516194 | 0.145 | Yes |
| rs769662 | 0.103 | Yes |
| rs77056528 | 0.149 | Yes |
| rs77101145 | 0.106 | Yes |
| rs77120325 | 0.225 | Yes |
| rs77151304 | 0.117 | Yes |
| rs77243303 | 0.144 | Yes |
| rs77522 | 0.105 | Yes |
| rs77704739 | 0.197 | Yes |
| rs77719426 | 0.179 | Yes |
| rs77808205 | 0.118 | Yes |
| rs77931950 | 0.129 | Yes |
| rs77960347 | 0.224 | Yes |
| rs78343493 | 0.110 | Yes |
| rs78620885 | 0.136 | Yes |
| rs78703482 | 0.109 | Yes |
| rs787488 | 0.103 | Yes |
| rs78769612 | 0.154 | Yes |
| rs78912080 | 0.121 | Yes |
| rs78948780 | 0.176 | Yes |
| rs79101008 | 0.106 | Yes |
| rs7956514 | 0.104 | Yes |
| rs7958316 | 0.138 | Yes |
| rs7970695 | 0.137 | Yes |
| rs79722469 | 0.139 | Yes |
| rs79896703 | 0.108 | Yes |
| rs7993752 | 0.102 | Yes |
| rs8008748 | 0.109 | Yes |
| rs8009347 | 0.103 | Yes |
| rs80272044 | 0.256 | Yes |
| rs8034216 | 0.103 | Yes |
| rs8040040 | 0.104 | Yes |
| rs8054651 | 0.114 | Yes |
| rs8059619 | 0.103 | Yes |
| rs8060025 | 0.103 | Yes |
| rs8077859 | 0.104 | Yes |
| rs8109532 | 0.103 | Yes |
| rs8126001 | 0.104 | Yes |
| rs8178824 | 0.157 | Yes |
| rs846879 | 0.103 | Yes |
| rs855679 | 0.115 | Yes |
| rs8978 | 0.104 | Yes |
| rs9266230 | 0.106 | Yes |
| rs9366639 | 0.111 | Yes |
| rs9368503 | 0.103 | Yes |
| rs9383643 | 0.105 | Yes |
| rs9388766 | 0.108 | Yes |
| rs9611454 | 0.108 | Yes |
| rs9738365 | 0.105 | Yes |
| rs9788721 | 0.102 | Yes |
| rs9826984 | 0.104 | Yes |
| rs984181 | 0.125 | Yes |
| rs9929143 | 0.114 | Yes |
| rs9951447 | 0.102 | Yes |
| rs9974178 | 0.103 | Yes |
| rs9988620 | 0.107 | Yes |
| rs10521222 | 0.141 | Yes |
| rs1183910 | 0.141 | Yes |
| rs12239046 | 0.107 | Yes |
| rs2794520 | 0.156 | Yes |
| rs2847281 | 0.103 | Yes |
| rs4129267 | 0.123 | Yes |
| rs4420065 | 0.145 | Yes |
| rs4705952 | 0.106 | Yes |
| rs6734238 | 0.113 | Yes |
| rs6901250 | 0.103 | Yes |
| Allele score | 1.250 | *No* |

| **Supplementary Table 5: Results of instrumental inequalities for MR models for the effect of triglyceride concentrations on coronary artery disease** | | |
| --- | --- | --- |
| **Proposed instrument** | **Value of instrumental inequalities** | **Do instrumental inequalities hold?** |
| rs38855 | 0.104 | Yes |
| rs3198697 | 0.103 | Yes |
| rs8077889 | 0.105 | Yes |
| rs10493326 | 0.112 | Yes |
| rs10513688 | 0.113 | Yes |
| rs10029254 | 0.107 | Yes |
| rs799160 | 0.110 | Yes |
| rs9693857 | 0.103 | Yes |
| rs4921914 | 0.109 | Yes |
| rs7033354 | 0.106 | Yes |
| rs1781930 | 0.104 | Yes |
| rs603446 | 0.109 | Yes |
| rs10861661 | 0.108 | Yes |
| rs1341267 | 0.106 | Yes |
| rs1035744 | 0.119 | Yes |
| rs749671 | 0.102 | Yes |
| rs9930333 | 0.102 | Yes |
| rs1688030 | 0.138 | Yes |
| rs3761445 | 0.110 | Yes |
| rs719726 | 0.104 | Yes |
| rs1495741 | 0.110 | Yes |
| rs7248104 | 0.103 | Yes |
| Allele score | 1.020 | *No* |

| **Supplementary Table 6: Results of instrumental inequalities for MR models for the effect of HDL-cholesterol concentrations on coronary artery disease** | | |
| --- | --- | --- |
| **Proposed instrument** | **Value of instrumental inequalities** | **Do instrumental inequalities hold?** |
| rs12145743 | 0.103 | Yes |
| rs4650994 | 0.103 | Yes |
| rs13326165 | 0.104 | Yes |
| rs2602836 | 0.105 | Yes |
| rs4917014 | 0.107 | Yes |
| rs17173637 | 0.107 | Yes |
| rs970548 | 0.109 | Yes |
| rs2923084 | 0.104 | Yes |
| rs12801636 | 0.106 | Yes |
| rs499974 | 0.110 | Yes |
| rs4983559 | 0.104 | Yes |
| rs16942887 | 0.113 | Yes |
| rs17695224 | 0.104 | Yes |
| rs181362 | 0.114 | Yes |
| rs4660293 | 0.110 | Yes |
| rs13107325 | 0.132 | Yes |
| rs6450176 | 0.107 | Yes |
| rs702485 | 0.102 | Yes |
| rs2293889 | 0.106 | Yes |
| rs11869286 | 0.105 | Yes |
| rs1689800 | 0.104 | Yes |
| rs4759375 | 0.112 | Yes |
| rs581080 | 0.111 | Yes |
| rs605066 | 0.103 | Yes |
| rs7255436 | 0.105 | Yes |
| rs737337 | 0.132 | Yes |
| rs838880 | 0.106 | Yes |
| rs12133576 | 0.104 | Yes |
| rs1689797 | 0.106 | Yes |
| rs6680658 | 0.104 | Yes |
| rs355838 | 0.107 | Yes |
| rs1047891 | 0.103 | Yes |
| rs2290547 | 0.106 | Yes |
| rs2240327 | 0.103 | Yes |
| rs6805251 | 0.102 | Yes |
| rs1482852 | 0.104 | Yes |
| rs10019888 | 0.106 | Yes |
| rs3822072 | 0.106 | Yes |
| rs4976033 | 0.103 | Yes |
| rs205262 | 0.107 | Yes |
| rs9491696 | 0.104 | Yes |
| rs12525163 | 0.103 | Yes |
| rs17286602 | 0.102 | Yes |
| rs10282707 | 0.106 | Yes |
| rs3996352 | 0.106 | Yes |
| rs4332136 | 0.148 | Yes |
| rs4871137 | 0.105 | Yes |
| rs4075205 | 0.104 | Yes |
| rs686030 | 0.113 | Yes |
| rs2472509 | 0.104 | Yes |
| rs2303975 | 0.109 | Yes |
| rs17788930 | 0.109 | Yes |
| rs11246602 | 0.115 | Yes |
| rs12226802 | 0.116 | Yes |
| rs7117842 | 0.104 | Yes |
| rs11045163 | 0.105 | Yes |
| rs2241210 | 0.106 | Yes |
| rs838876 | 0.107 | Yes |
| rs10773105 | 0.106 | Yes |
| rs931992 | 0.105 | Yes |
| rs4148005 | 0.104 | Yes |
| rs4969178 | 0.109 | Yes |
| rs4939883 | 0.119 | Yes |
| rs11660468 | 0.110 | Yes |
| rs952044 | 0.107 | Yes |
| rs2278236 | 0.106 | Yes |
| rs103294 | 0.112 | Yes |
| rs1121980 | 0.107 | Yes |
| rs12328675 | 0.117 | Yes |
| rs2954029 | 0.108 | Yes |
| rs3136441 | 0.114 | Yes |
| rs386000 | 0.110 | Yes |
| rs7134375 | 0.105 | Yes |
| rs7941030 | 0.105 | Yes |
| rs2606736 | 0.102 | Yes |
| rs4129767 | 0.107 | Yes |
| rs4731702 | 0.106 | Yes |
| rs4765127 | 0.109 | Yes |
| rs7134594 | 0.106 | Yes |
| rs1936800 | 0.104 | Yes |
| rs4142995 | 0.106 | Yes |
| rs4148008 | 0.104 | Yes |
| Allele score | 1.053 | *No* |

| **Supplementary Table 7: Results of the instrumental inequalities applied to MR models for the effect of LDL-cholesterol concentrations on coronary artery disease** | | |
| --- | --- | --- |
| **Proposed instrument** | **Value of instrumental inequalities** | **Do instrumental inequalities hold?** |
| rs1010167 | 0.103 | Yes |
| rs267733 | 0.107 | Yes |
| rs11563251 | 0.111 | Yes |
| rs7640978 | 0.121 | Yes |
| rs1564348 | 0.117 | Yes |
| rs12670798 | 0.110 | Yes |
| rs10102164 | 0.108 | Yes |
| rs3780181 | 0.130 | Yes |
| rs1169288 | 0.110 | Yes |
| rs4942486 | 0.106 | Yes |
| rs8017377 | 0.106 | Yes |
| rs364585 | 0.102 | Yes |
| rs2328223 | 0.107 | Yes |
| rs5763662 | 0.143 | Yes |
| rs10903129 | 0.105 | Yes |
| rs2587534 | 0.105 | Yes |
| rs515135 | 0.130 | Yes |
| rs6544713 | 0.120 | Yes |
| rs4148218 | 0.110 | Yes |
| rs2710642 | 0.103 | Yes |
| rs17508045 | 0.117 | Yes |
| rs2030746 | 0.103 | Yes |
| rs16831243 | 0.120 | Yes |
| rs1250229 | 0.106 | Yes |
| rs9875338 | 0.103 | Yes |
| rs4530754 | 0.104 | Yes |
| rs1800562 | 0.149 | Yes |
| rs2297374 | 0.106 | Yes |
| rs2073547 | 0.110 | Yes |
| rs217386 | 0.104 | Yes |
| rs2737252 | 0.105 | Yes |
| rs7832643 | 0.103 | Yes |
| rs8176720 | 0.106 | Yes |
| rs579459 | 0.113 | Yes |
| rs10832962 | 0.116 | Yes |
| rs11220462 | 0.114 | Yes |
| rs2000999 | 0.118 | Yes |
| rs314253 | 0.104 | Yes |
| rs6511720 | 0.142 | Yes |
| rs688 | 0.110 | Yes |
| rs6859 | 0.116 | Yes |
| rs492602 | 0.109 | Yes |
| rs7264396 | 0.112 | Yes |
| rs6016381 | 0.105 | Yes |
| rs6065311 | 0.104 | Yes |
| rs2479409 | 0.110 | Yes |
| rs2642442 | 0.103 | Yes |
| rs629301 | 0.128 | Yes |
| rs4299376 | 0.121 | Yes |
| rs6818397 | 0.102 | Yes |
| rs3177928 | 0.114 | Yes |
| rs3757354 | 0.111 | Yes |
| rs11136341 | 0.104 | Yes |
| rs1801689 | 0.137 | Yes |
| rs1998013 | 0.506 | Yes |
| rs6603981 | 0.104 | Yes |
| rs903319 | 0.104 | Yes |
| rs2287623 | 0.103 | Yes |
| rs17345563 | 0.110 | Yes |
| rs7703051 | 0.114 | Yes |
| rs2294261 | 0.107 | Yes |
| rs17789218 | 0.104 | Yes |
| rs868943 | 0.103 | Yes |
| rs2326077 | 0.109 | Yes |
| rs174532 | 0.109 | Yes |
| rs6489818 | 0.115 | Yes |
| rs1186380 | 0.113 | Yes |
| rs2288002 | 0.103 | Yes |
| rs4791641 | 0.102 | Yes |
| rs7225700 | 0.104 | Yes |
| rs10410 | 0.113 | Yes |
| rs10910490 | 0.106 | Yes |
| rs11102964 | 0.114 | Yes |
| rs11206510 | 0.113 | Yes |
| rs11206514 | 0.112 | Yes |
| rs11485618 | 0.107 | Yes |
| rs11581665 | 0.111 | Yes |
| rs11583974 | 0.114 | Yes |
| rs11591147 | 0.348 | Yes |
| rs12066643 | 0.103 | Yes |
| rs12127701 | 0.119 | Yes |
| rs12129277 | 0.107 | Yes |
| rs12410656 | 0.114 | Yes |
| rs1278286 | 0.112 | Yes |
| rs1337247 | 0.125 | Yes |
| rs13375691 | 0.108 | Yes |
| rs1386585 | 0.104 | Yes |
| rs1475701 | 0.115 | Yes |
| rs17035630 | 0.108 | Yes |
| rs17035665 | 0.109 | Yes |
| rs17647543 | 0.123 | Yes |
| rs1874776 | 0.106 | Yes |
| rs207145 | 0.106 | Yes |
| rs2247213 | 0.105 | Yes |
| rs2479394 | 0.105 | Yes |
| rs2647281 | 0.109 | Yes |
| rs413380 | 0.137 | Yes |
| rs413582 | 0.107 | Yes |
| rs4847221 | 0.113 | Yes |
| rs4927207 | 0.112 | Yes |
| rs4970712 | 0.104 | Yes |
| rs572512 | 0.104 | Yes |
| rs585131 | 0.107 | Yes |
| rs585362 | 0.119 | Yes |
| rs650985 | 0.114 | Yes |
| rs6662286 | 0.113 | Yes |
| rs6689614 | 0.112 | Yes |
| rs72703204 | 0.133 | Yes |
| rs7512480 | 0.105 | Yes |
| rs7544735 | 0.104 | Yes |
| rs7552841 | 0.109 | Yes |
| rs10195252 | 0.106 | Yes |
| rs10208987 | 0.113 | Yes |
| rs1025447 | 0.105 | Yes |
| rs10490626 | 0.116 | Yes |
| rs11096689 | 0.115 | Yes |
| rs11679386 | 0.118 | Yes |
| rs11685356 | 0.119 | Yes |
| rs12471982 | 0.114 | Yes |
| rs12691202 | 0.127 | Yes |
| rs12720796 | 0.129 | Yes |
| rs12720842 | 0.128 | Yes |
| rs13027175 | 0.120 | Yes |
| rs1534420 | 0.104 | Yes |
| rs17398765 | 0.128 | Yes |
| rs2194562 | 0.107 | Yes |
| rs312049 | 0.111 | Yes |
| rs3791981 | 0.135 | Yes |
| rs4148177 | 0.108 | Yes |
| rs492399 | 0.167 | Yes |
| rs4953023 | 0.125 | Yes |
| rs4988235 | 0.117 | Yes |
| rs6413458 | 0.127 | Yes |
| rs6547409 | 0.141 | Yes |
| rs6725189 | 0.108 | Yes |
| rs6729410 | 0.112 | Yes |
| rs6739502 | 0.108 | Yes |
| rs6754295 | 0.116 | Yes |
| rs6756743 | 0.144 | Yes |
| rs6759321 | 0.118 | Yes |
| rs75279593 | 0.127 | Yes |
| rs7567653 | 0.134 | Yes |
| rs780093 | 0.111 | Yes |
| rs11709504 | 0.109 | Yes |
| rs17819328 | 0.103 | Yes |
| rs10069744 | 0.116 | Yes |
| rs10515198 | 0.121 | Yes |
| rs10515214 | 0.108 | Yes |
| rs12916 | 0.114 | Yes |
| rs16872670 | 0.119 | Yes |
| rs3857388 | 0.111 | Yes |
| rs4361493 | 0.105 | Yes |
| rs4703642 | 0.105 | Yes |
| rs4704231 | 0.112 | Yes |
| rs4704810 | 0.102 | Yes |
| rs6873053 | 0.110 | Yes |
| rs7717505 | 0.111 | Yes |
| rs7727150 | 0.103 | Yes |
| rs10455872 | 0.125 | Yes |
| rs117733303 | 0.151 | Yes |
| rs12208357 | 0.128 | Yes |
| rs1367211 | 0.107 | Yes |
| rs1408272 | 0.146 | Yes |
| rs16891156 | 0.126 | Yes |
| rs2327951 | 0.112 | Yes |
| rs2621321 | 0.109 | Yes |
| rs3120139 | 0.110 | Yes |
| rs3798180 | 0.108 | Yes |
| rs3798221 | 0.108 | Yes |
| rs389883 | 0.102 | Yes |
| rs3918291 | 0.168 | Yes |
| rs446218 | 0.103 | Yes |
| rs461473 | 0.110 | Yes |
| rs6909746 | 0.102 | Yes |
| rs6917747 | 0.114 | Yes |
| rs6935921 | 0.108 | Yes |
| rs7774197 | 0.121 | Yes |
| rs9457843 | 0.109 | Yes |
| rs6461566 | 0.105 | Yes |
| rs10102352 | 0.106 | Yes |
| rs13277801 | 0.109 | Yes |
| rs2081687 | 0.109 | Yes |
| rs2980875 | 0.112 | Yes |
| rs4360309 | 0.103 | Yes |
| rs4592055 | 0.111 | Yes |
| rs11244084 | 0.123 | Yes |
| rs11795315 | 0.106 | Yes |
| rs4489379 | 0.108 | Yes |
| rs630014 | 0.103 | Yes |
| rs7030248 | 0.104 | Yes |
| rs8176693 | 0.122 | Yes |
| rs2419604 | 0.104 | Yes |
| rs10893493 | 0.106 | Yes |
| rs10893499 | 0.112 | Yes |
| rs10893505 | 0.115 | Yes |
| rs11600380 | 0.116 | Yes |
| rs12294259 | 0.115 | Yes |
| rs174476 | 0.107 | Yes |
| rs174583 | 0.106 | Yes |
| rs180326 | 0.105 | Yes |
| rs2075290 | 0.116 | Yes |
| rs2845573 | 0.114 | Yes |
| rs508487 | 0.124 | Yes |
| rs582037 | 0.108 | Yes |
| rs10850003 | 0.116 | Yes |
| rs11065987 | 0.103 | Yes |
| rs11066028 | 0.108 | Yes |
| rs17630235 | 0.103 | Yes |
| rs2708101 | 0.104 | Yes |
| rs3184504 | 0.104 | Yes |
| rs657197 | 0.111 | Yes |
| rs7953150 | 0.108 | Yes |
| rs12448528 | 0.106 | Yes |
| rs12931964 | 0.112 | Yes |
| rs1864163 | 0.107 | Yes |
| rs217181 | 0.107 | Yes |
| rs247616 | 0.108 | Yes |
| rs7197967 | 0.113 | Yes |
| rs8044335 | 0.104 | Yes |
| rs8044476 | 0.109 | Yes |
| rs8060878 | 0.103 | Yes |
| rs9302635 | 0.123 | Yes |
| rs2886232 | 0.119 | Yes |
| rs6504872 | 0.105 | Yes |
| rs7206971 | 0.105 | Yes |
| rs8070463 | 0.104 | Yes |
| rs10402271 | 0.120 | Yes |
| rs10403668 | 0.108 | Yes |
| rs10419669 | 0.115 | Yes |
| rs10422616 | 0.106 | Yes |
| rs10460181 | 0.119 | Yes |
| rs1048699 | 0.124 | Yes |
| rs11668536 | 0.104 | Yes |
| rs11669133 | 0.119 | Yes |
| rs11881156 | 0.112 | Yes |
| rs12150984 | 0.105 | Yes |
| rs12721109 | 0.349 | Yes |
| rs1529711 | 0.109 | Yes |
| rs1531517 | 0.195 | Yes |
| rs157580 | 0.118 | Yes |
| rs1594895 | 0.111 | Yes |
| rs16979372 | 0.160 | Yes |
| rs16996148 | 0.167 | Yes |
| rs17677316 | 0.110 | Yes |
| rs17800760 | 0.116 | Yes |
| rs1799898 | 0.105 | Yes |
| rs2075650 | 0.155 | Yes |
| rs2287019 | 0.105 | Yes |
| rs2315025 | 0.110 | Yes |
| rs2927477 | 0.132 | Yes |
| rs2965101 | 0.120 | Yes |
| rs2965157 | 0.192 | Yes |
| rs2965174 | 0.109 | Yes |
| rs2972564 | 0.105 | Yes |
| rs3208856 | 0.223 | Yes |
| rs36005514 | 0.125 | Yes |
| rs376642 | 0.104 | Yes |
| rs3786721 | 0.107 | Yes |
| rs3786722 | 0.109 | Yes |
| rs379309 | 0.105 | Yes |
| rs387976 | 0.117 | Yes |
| rs445925 | 0.300 | Yes |
| rs4803760 | 0.140 | Yes |
| rs4803766 | 0.106 | Yes |
| rs5158 | 0.117 | Yes |
| rs5742911 | 0.109 | Yes |
| rs59325138 | 0.113 | Yes |
| rs714948 | 0.115 | Yes |
| rs7251031 | 0.109 | Yes |
| rs7252981 | 0.105 | Yes |
| rs7255743 | 0.208 | Yes |
| rs73015030 | 0.154 | Yes |
| rs73045960 | 0.196 | Yes |
| rs77301115 | 0.148 | Yes |
| rs8102380 | 0.102 | Yes |
| rs8103315 | 0.115 | Yes |
| rs8108762 | 0.109 | Yes |
| rs892114 | 0.112 | Yes |
| rs926054 | 0.120 | Yes |
| rs9973305 | 0.125 | Yes |
| rs2745865 | 0.110 | Yes |
| rs2865507 | 0.103 | Yes |
| rs2902940 | 0.107 | Yes |
| rs4142393 | 0.103 | Yes |
| rs4812494 | 0.105 | Yes |
| rs6124309 | 0.104 | Yes |
| rs742748 | 0.104 | Yes |
| rs4253772 | 0.106 | Yes |
| rs4253776 | 0.123 | Yes |
| rs76733602 | 0.117 | Yes |
| rs68160747 | 0.123 | Yes |
| rs72768351 | 0.134 | Yes |
| rs4703665 | 0.104 | Yes |
| rs6877840 | 0.113 | Yes |
| rs17244939 | 0.129 | Yes |
| rs5908 | 0.139 | Yes |
| rs74695562 | 0.113 | Yes |
| rs7706933 | 0.174 | Yes |
| rs3804231 | 0.118 | Yes |
| rs80324692 | 0.108 | Yes |
| rs75944831 | 0.177 | Yes |
| rs62366598 | 0.108 | Yes |
| rs11206479 | 0.102 | Yes |
| rs10218716 | 0.102 | Yes |
| rs2500340 | 0.108 | Yes |
| rs943645 | 0.102 | Yes |
| rs12093385 | 0.105 | Yes |
| rs12075602 | 0.112 | Yes |
| rs17111474 | 0.101 | Yes |
| rs2479398 | 0.104 | Yes |
| rs2864123 | 0.111 | Yes |
| rs2479396 | 0.104 | Yes |
| rs17111483 | 0.136 | Yes |
| rs17111490 | 0.109 | Yes |
| rs12739979 | 0.107 | Yes |
| rs11810371 | 0.147 | Yes |
| rs77406753 | 0.105 | Yes |
| rs2479406 | 0.131 | Yes |
| rs28385701 | 0.176 | Yes |
| rs74700387 | 0.140 | Yes |
| rs28385708 | 0.125 | Yes |
| rs11800243 | 0.114 | Yes |
| rs12067569 | 0.128 | Yes |
| rs10493176 | 0.123 | Yes |
| rs17111657 | 0.161 | Yes |
| rs960502 | 0.125 | Yes |
| rs12138592 | 0.112 | Yes |
| rs80355736 | 0.156 | Yes |
| rs117175530 | 0.156 | Yes |
| rs4724302 | 0.120 | Yes |
| rs73107405 | 0.128 | Yes |
| rs113496141 | 0.122 | Yes |
| rs6956388 | 0.113 | Yes |
| rs10234070 | 0.111 | Yes |
| rs76236858 | 0.152 | Yes |
| rs76331929 | 0.132 | Yes |
| rs217437 | 0.102 | Yes |
| rs35349497 | 0.114 | Yes |
| rs41279627 | 0.114 | Yes |
| rs113223818 | 0.130 | Yes |
| rs56243746 | 0.130 | Yes |
| rs117843715 | 0.150 | Yes |
| rs117623941 | 0.153 | Yes |
| rs2008036 | 0.110 | Yes |
| rs77516609 | 0.111 | Yes |
| rs7792931 | 0.135 | Yes |
| rs118187778 | 0.120 | Yes |
| rs79836087 | 0.125 | Yes |
| rs76446485 | 0.135 | Yes |
| rs80169634 | 0.113 | Yes |
| rs76203970 | 0.149 | Yes |
| rs114776298 | 0.108 | Yes |
| rs1801695 | 0.117 | Yes |
| rs1801700 | 0.117 | Yes |
| rs72653053 | 0.163 | Yes |
| rs62122522 | 0.115 | Yes |
| rs114368165 | 0.183 | Yes |
| rs115071638 | 0.150 | Yes |
| rs12470778 | 0.109 | Yes |
| rs312966 | 0.104 | Yes |
| rs113588790 | 0.123 | Yes |
| rs72782175 | 0.169 | Yes |
| rs1801703 | 0.167 | Yes |
| rs1801699 | 0.167 | Yes |
| rs79112951 | 0.111 | Yes |
| rs17519079 | 0.111 | Yes |
| rs1263149 | 0.102 | Yes |
| rs34552724 | 0.140 | Yes |
| rs78296522 | 0.123 | Yes |
| rs74773964 | 0.140 | Yes |
| rs633389 | 0.148 | Yes |
| rs5110 | 0.109 | Yes |
| rs11216164 | 0.103 | Yes |
| rs548638 | 0.102 | Yes |
| rs11216169 | 0.107 | Yes |
| rs543819 | 0.118 | Yes |
| rs17174502 | 0.106 | Yes |
| rs7943309 | 0.122 | Yes |
| rs7106782 | 0.134 | Yes |
| rs17678136 | 0.112 | Yes |
| rs13345127 | 0.114 | Yes |
| rs2072382 | 0.117 | Yes |
| rs2421198 | 0.104 | Yes |
| rs17242346 | 0.131 | Yes |
| rs12981050 | 0.122 | Yes |
| rs17242367 | 0.111 | Yes |
| rs17248748 | 0.174 | Yes |
| rs2569556 | 0.125 | Yes |
| rs6413503 | 0.159 | Yes |
| rs3826810 | 0.120 | Yes |
| rs72658879 | 0.122 | Yes |
| rs4804146 | 0.110 | Yes |
| rs892113 | 0.132 | Yes |
| rs4804149 | 0.104 | Yes |
| rs11880059 | 0.138 | Yes |
| rs17616661 | 0.108 | Yes |
| rs4804150 | 0.105 | Yes |
| rs4804576 | 0.164 | Yes |
| rs12027135 | 0.106 | Yes |
| rs514230 | 0.105 | Yes |
| rs2072183 | 0.112 | Yes |
| rs6029526 | 0.104 | Yes |
| Allele score | 1.200 | *No* |

**S9. Supplementary tables 8-13: Results of the instrumental inequalities for each SNP proposed as an instrument in inverse-probability weighted pseudo populations**

| **Supplemental Table 8: Results of instrumental inequalities applied to MR models for the effect of vitamin D concentration on coronary artery disease. in pseudo population inverse probability weighted for 10 principal components** | | |  |
| --- | --- | --- | --- |
| **Proposed instrument** | **Value of instrumental inequalities** | **Do instrumental inequalities hold?** | |
| rs12785878 | 0.120 | Yes | |
| rs6013897 | 0.107 | Yes | |
| rs10741657 | 0.143 | Yes | |
| rs12794714 | 0.135 | Yes | |
| rs116970203 | 0.363 | Yes | |
| rs3755967 | 0.140 | Yes | |
| rs17216707 | 0.111 | Yes | |
| rs10745742 | 0.106 | Yes | |
| rs8018720 | 0.123 | Yes | |
| rs117913124 | 0.345 | Yes | |
| rs2282679 | 0.140 | Yes | |
| rs7944926 | 0.120 | Yes | |
| rs11234027 | 0.125 | Yes | |
| rs4588 | 0.140 | Yes | |
| rs4423214 | 0.120 | Yes | |
| Allele score | 0.599 | Yes | |

| **Supplementary Table 9: Results of the instrumental inequalities for MR models for the effect of alcohol consumption on coronary artery disease in pseudo populations inverse probability weighted for 10 principal components** | | |
| --- | --- | --- |
| **Proposed instrument** | **Value of instrumental inequalities** | **Do instrumental inequalities hold?** |
| rs11039429 | 0.259 | Yes |
| rs11787216 | 0.263 | Yes |
| rs11940694 | 0.260 | Yes |
| rs13102973 | 0.259 | Yes |
| rs13231886 | 0.259 | Yes |
| rs13390019 | 0.261 | Yes |
| rs17097556 | 0.259 | Yes |
| rs17690703 | 0.266 | Yes |
| rs1788030 | 0.260 | Yes |
| rs1893659 | 0.260 | Yes |
| rs2159935 | 0.259 | Yes |
| rs363096 | 0.260 | Yes |
| rs4726481 | 0.260 | Yes |
| rs540606 | 0.261 | Yes |
| rs571312 | 0.260 | Yes |
| rs58905411 | 0.261 | Yes |
| rs6016781 | 0.260 | Yes |
| rs650558 | 0.261 | Yes |
| rs7428430 | 0.261 | Yes |
| rs838145 | 0.259 | Yes |
| rs9372625 | 0.261 | Yes |
| rs9842406 | 0.259 | Yes |
| rs9923768 | 0.259 | Yes |
| rs145452708 | 0.290 | Yes |
| rs193099203 | 0.268 | Yes |
| rs29001570 | 0.379 | Yes |
| rs9841829 | 0.261 | Yes |
| rs1229984 | 0.265 | Yes |
| Allele Score | 1.226 | *No* |

| **Supplementary Table 10: Results of the instrumental inequalities for MR models for the effect of c-reactive protein on coronary artery disease in pseudo populations inverse probability weighted for 10 principal components** | | |
| --- | --- | --- |
| **Proposed instrument** | **Value of instrumental inequalities** | **Do instrumental inequalities hold?** |
| rs10027182 | 0.105 | Yes |
| rs10083137 | 0.123 | Yes |
| rs10095930 | 0.105 | Yes |
| rs10106298 | 0.105 | Yes |
| rs10203386 | 0.104 | Yes |
| rs1037170 | 0.106 | Yes |
| rs1045075 | 0.103 | Yes |
| rs10453441 | 0.102 | Yes |
| rs10490871 | 0.104 | Yes |
| rs1052067 | 0.104 | Yes |
| rs1056441 | 0.105 | Yes |
| rs10752652 | 0.103 | Yes |
| rs10755296 | 0.105 | Yes |
| rs10760691 | 0.104 | Yes |
| rs10779035 | 0.103 | Yes |
| rs10794644 | 0.104 | Yes |
| rs10802496 | 0.110 | Yes |
| rs10806385 | 0.103 | Yes |
| rs10810455 | 0.104 | Yes |
| rs10831676 | 0.103 | Yes |
| rs10848035 | 0.106 | Yes |
| rs10849773 | 0.139 | Yes |
| rs10864086 | 0.106 | Yes |
| rs10924372 | 0.103 | Yes |
| rs10957159 | 0.108 | Yes |
| rs11000760 | 0.104 | Yes |
| rs11001477 | 0.106 | Yes |
| rs11012732 | 0.103 | Yes |
| rs11047139 | 0.108 | Yes |
| rs11057214 | 0.114 | Yes |
| rs11065050 | 0.125 | Yes |
| rs11078597 | 0.105 | Yes |
| rs11083166 | 0.103 | Yes |
| rs111237020 | 0.117 | Yes |
| rs11128901 | 0.103 | Yes |
| rs111363146 | 0.109 | Yes |
| rs11138311 | 0.110 | Yes |
| rs111435864 | 0.112 | Yes |
| rs111570031 | 0.121 | Yes |
| rs11158975 | 0.105 | Yes |
| rs11210869 | 0.103 | Yes |
| rs11222703 | 0.102 | Yes |
| rs11227395 | 0.105 | Yes |
| rs112283362 | 0.174 | Yes |
| rs11229252 | 0.112 | Yes |
| rs11231161 | 0.104 | Yes |
| rs11246574 | 0.109 | Yes |
| rs112661041 | 0.111 | Yes |
| rs112689575 | 0.129 | Yes |
| rs112750178 | 0.105 | Yes |
| rs114451718 | 0.191 | Yes |
| rs114574084 | 0.134 | Yes |
| rs114697636 | 0.125 | Yes |
| rs114733917 | 0.130 | Yes |
| rs114947103 | 0.106 | Yes |
| rs115023714 | 0.143 | Yes |
| rs115093996 | 0.186 | Yes |
| rs115178836 | 0.198 | Yes |
| rs115273594 | 0.250 | Yes |
| rs115462819 | 0.126 | Yes |
| rs11577023 | 0.104 | Yes |
| rs116073407 | 0.604 | Yes |
| rs116424768 | 0.169 | Yes |
| rs11648192 | 0.103 | Yes |
| rs11648261 | 0.106 | Yes |
| rs11652684 | 0.106 | Yes |
| rs11666245 | 0.114 | Yes |
| rs11667234 | 0.130 | Yes |
| rs11672020 | 0.104 | Yes |
| rs116804861 | 0.104 | Yes |
| rs11681145 | 0.104 | Yes |
| rs11682186 | 0.104 | Yes |
| rs11693537 | 0.104 | Yes |
| rs11693697 | 0.105 | Yes |
| rs116971887 | 0.141 | Yes |
| rs11708067 | 0.103 | Yes |
| rs11711864 | 0.101 | Yes |
| rs117211511 | 0.128 | Yes |
| rs117264457 | 0.137 | Yes |
| rs117368206 | 0.163 | Yes |
| rs117548270 | 0.176 | Yes |
| rs11767338 | 0.106 | Yes |
| rs117695016 | 0.132 | Yes |
| rs11772703 | 0.103 | Yes |
| rs117783785 | 0.168 | Yes |
| rs117803005 | 0.123 | Yes |
| rs117849038 | 0.204 | Yes |
| rs11928797 | 0.107 | Yes |
| rs12054057 | 0.106 | Yes |
| rs12055445 | 0.103 | Yes |
| rs12077265 | 0.120 | Yes |
| rs12132412 | 0.104 | Yes |
| rs12138486 | 0.107 | Yes |
| rs12140153 | 0.106 | Yes |
| rs12141189 | 0.106 | Yes |
| rs12211604 | 0.105 | Yes |
| rs12231235 | 0.107 | Yes |
| rs12280456 | 0.111 | Yes |
| rs12357890 | 0.103 | Yes |
| rs123698 | 0.105 | Yes |
| rs12425953 | 0.123 | Yes |
| rs12426126 | 0.113 | Yes |
| rs12443906 | 0.104 | Yes |
| rs12448756 | 0.108 | Yes |
| rs12466485 | 0.108 | Yes |
| rs12516176 | 0.105 | Yes |
| rs12517168 | 0.103 | Yes |
| rs12601665 | 0.104 | Yes |
| rs12605964 | 0.103 | Yes |
| rs12620844 | 0.104 | Yes |
| rs12679106 | 0.102 | Yes |
| rs12712928 | 0.108 | Yes |
| rs12714415 | 0.107 | Yes |
| rs12716871 | 0.102 | Yes |
| rs12735458 | 0.150 | Yes |
| rs12828693 | 0.107 | Yes |
| rs1290898 | 0.112 | Yes |
| rs1292067 | 0.105 | Yes |
| rs12933292 | 0.104 | Yes |
| rs12966114 | 0.102 | Yes |
| rs12992747 | 0.105 | Yes |
| rs12992995 | 0.104 | Yes |
| rs13021948 | 0.140 | Yes |
| rs13058527 | 0.106 | Yes |
| rs13066686 | 0.103 | Yes |
| rs13081778 | 0.106 | Yes |
| rs13106834 | 0.103 | Yes |
| rs13108218 | 0.103 | Yes |
| rs13227497 | 0.110 | Yes |
| rs1338721 | 0.104 | Yes |
| rs13416992 | 0.103 | Yes |
| rs1353792 | 0.104 | Yes |
| rs139136389 | 0.171 | Yes |
| rs139460294 | 0.153 | Yes |
| rs139952834 | 0.167 | Yes |
| rs141094656 | 0.152 | Yes |
| rs1412444 | 0.107 | Yes |
| rs141651106 | 0.127 | Yes |
| rs141737681 | 0.370 | Yes |
| rs142603322 | 0.144 | Yes |
| rs143273561 | 0.150 | Yes |
| rs1441043 | 0.106 | Yes |
| rs1441169 | 0.105 | Yes |
| rs144314390 | 0.319 | Yes |
| rs144398877 | 0.139 | Yes |
| rs144970957 | 0.168 | Yes |
| rs145987962 | 0.115 | Yes |
| rs145990041 | 0.218 | Yes |
| rs146342974 | 0.154 | Yes |
| rs146396094 | 0.164 | Yes |
| rs1476698 | 0.104 | Yes |
| rs148059420 | 0.138 | Yes |
| rs148104667 | 0.715 | Yes |
| rs148303016 | 0.128 | Yes |
| rs148770227 | 0.137 | Yes |
| rs148933445 | 0.147 | Yes |
| rs1490384 | 0.106 | Yes |
| rs1492914 | 0.105 | Yes |
| rs1495017 | 0.104 | Yes |
| rs149523036 | 0.233 | Yes |
| rs1495745 | 0.103 | Yes |
| rs149647262 | 0.112 | Yes |
| rs150365073 | 0.158 | Yes |
| rs150377363 | 0.162 | Yes |
| rs150543164 | 0.121 | Yes |
| rs150832462 | 0.108 | Yes |
| rs150844304 | 0.140 | Yes |
| rs150852956 | 0.136 | Yes |
| rs151132887 | 0.153 | Yes |
| rs1545536 | 0.106 | Yes |
| rs1546721 | 0.102 | Yes |
| rs1570868 | 0.105 | Yes |
| rs157512 | 0.106 | Yes |
| rs1635852 | 0.104 | Yes |
| rs16835819 | 0.160 | Yes |
| rs16842320 | 0.130 | Yes |
| rs1693456 | 0.102 | Yes |
| rs17040284 | 0.105 | Yes |
| rs17105232 | 0.104 | Yes |
| rs17138476 | 0.107 | Yes |
| rs17308476 | 0.109 | Yes |
| rs1736057 | 0.106 | Yes |
| rs17366139 | 0.117 | Yes |
| rs17417252 | 0.103 | Yes |
| rs174373 | 0.106 | Yes |
| rs17616063 | 0.143 | Yes |
| rs17626434 | 0.102 | Yes |
| rs17854914 | 0.110 | Yes |
| rs178835 | 0.103 | Yes |
| rs1800693 | 0.103 | Yes |
| rs1800919 | 0.108 | Yes |
| rs1800973 | 0.113 | Yes |
| rs1801272 | 0.119 | Yes |
| rs180686303 | 0.113 | Yes |
| rs182638776 | 0.108 | Yes |
| rs183553104 | 0.149 | Yes |
| rs184334219 | 0.179 | Yes |
| rs185575165 | 0.108 | Yes |
| rs1880241 | 0.105 | Yes |
| rs1886008 | 0.111 | Yes |
| rs1905505 | 0.104 | Yes |
| rs190712692 | 0.115 | Yes |
| rs1933736 | 0.104 | Yes |
| rs1968109 | 0.103 | Yes |
| rs1969026 | 0.127 | Yes |
| rs1975190 | 0.113 | Yes |
| rs1985157 | 0.104 | Yes |
| rs198851 | 0.105 | Yes |
| rs2011689 | 0.103 | Yes |
| rs2030291 | 0.103 | Yes |
| rs204914 | 0.122 | Yes |
| rs2054434 | 0.105 | Yes |
| rs2075803 | 0.103 | Yes |
| rs209474 | 0.103 | Yes |
| rs2107135 | 0.104 | Yes |
| rs2110944 | 0.104 | Yes |
| rs2154384 | 0.143 | Yes |
| rs2161037 | 0.104 | Yes |
| rs2161374 | 0.103 | Yes |
| rs2201637 | 0.116 | Yes |
| rs2207132 | 0.135 | Yes |
| rs2233278 | 0.113 | Yes |
| rs2239222 | 0.107 | Yes |
| rs2239704 | 0.104 | Yes |
| rs2250010 | 0.109 | Yes |
| rs2256027 | 0.123 | Yes |
| rs2265189 | 0.102 | Yes |
| rs2269434 | 0.105 | Yes |
| rs2269841 | 0.105 | Yes |
| rs2301275 | 0.103 | Yes |
| rs2302758 | 0.121 | Yes |
| rs2311597 | 0.103 | Yes |
| rs2378253 | 0.102 | Yes |
| rs2393794 | 0.109 | Yes |
| rs2432195 | 0.108 | Yes |
| rs245768 | 0.105 | Yes |
| rs2472682 | 0.103 | Yes |
| rs2494097 | 0.106 | Yes |
| rs2542170 | 0.105 | Yes |
| rs2627646 | 0.114 | Yes |
| rs263013 | 0.103 | Yes |
| rs2638315 | 0.108 | Yes |
| rs2668244 | 0.106 | Yes |
| rs2681780 | 0.105 | Yes |
| rs2700938 | 0.106 | Yes |
| rs2721953 | 0.103 | Yes |
| rs2740479 | 0.105 | Yes |
| rs2808474 | 0.105 | Yes |
| rs2823674 | 0.103 | Yes |
| rs28361325 | 0.103 | Yes |
| rs2836882 | 0.109 | Yes |
| rs28399607 | 0.118 | Yes |
| rs28601761 | 0.104 | Yes |
| rs288183 | 0.104 | Yes |
| rs2882485 | 0.102 | Yes |
| rs28929474 | 0.143 | Yes |
| rs2927447 | 0.116 | Yes |
| rs2957664 | 0.102 | Yes |
| rs2961111 | 0.104 | Yes |
| rs2965164 | 0.104 | Yes |
| rs2970901 | 0.103 | Yes |
| rs2980512 | 0.103 | Yes |
| rs3014201 | 0.103 | Yes |
| rs301804 | 0.103 | Yes |
| rs3027003 | 0.137 | Yes |
| rs3095122 | 0.103 | Yes |
| rs3125326 | 0.103 | Yes |
| rs3134133 | 0.105 | Yes |
| rs33951980 | 0.122 | Yes |
| rs340005 | 0.107 | Yes |
| rs34061534 | 0.144 | Yes |
| rs34284056 | 0.104 | Yes |
| rs34415150 | 0.112 | Yes |
| rs34761529 | 0.105 | Yes |
| rs34874378 | 0.128 | Yes |
| rs352126 | 0.104 | Yes |
| rs35582280 | 0.103 | Yes |
| rs35650976 | 0.104 | Yes |
| rs35764600 | 0.102 | Yes |
| rs35860194 | 0.180 | Yes |
| rs35881303 | 0.103 | Yes |
| rs36049560 | 0.105 | Yes |
| rs3733892 | 0.104 | Yes |
| rs3736164 | 0.104 | Yes |
| rs3746778 | 0.104 | Yes |
| rs3750310 | 0.103 | Yes |
| rs3751143 | 0.106 | Yes |
| rs3768321 | 0.108 | Yes |
| rs3774063 | 0.114 | Yes |
| rs3785568 | 0.105 | Yes |
| rs3789988 | 0.142 | Yes |
| rs3794204 | 0.112 | Yes |
| rs380825 | 0.102 | Yes |
| rs3808348 | 0.109 | Yes |
| rs3826559 | 0.103 | Yes |
| rs385417 | 0.105 | Yes |
| rs3856270 | 0.103 | Yes |
| rs3865444 | 0.104 | Yes |
| rs4018180 | 0.117 | Yes |
| rs4084164 | 0.103 | Yes |
| rs41290108 | 0.121 | Yes |
| rs4148155 | 0.104 | Yes |
| rs41523449 | 0.109 | Yes |
| rs420957 | 0.104 | Yes |
| rs4239504 | 0.105 | Yes |
| rs424539 | 0.103 | Yes |
| rs4255379 | 0.115 | Yes |
| rs4266763 | 0.106 | Yes |
| rs429358 | 0.230 | Yes |
| rs4411129 | 0.107 | Yes |
| rs4431051 | 0.104 | Yes |
| rs4499304 | 0.102 | Yes |
| rs4506495 | 0.108 | Yes |
| rs4516268 | 0.105 | Yes |
| rs4535048 | 0.106 | Yes |
| rs4641306 | 0.126 | Yes |
| rs4658403 | 0.107 | Yes |
| rs469882 | 0.112 | Yes |
| rs4704780 | 0.113 | Yes |
| rs4714508 | 0.103 | Yes |
| rs4755720 | 0.103 | Yes |
| rs4764939 | 0.104 | Yes |
| rs4766960 | 0.106 | Yes |
| rs4767938 | 0.104 | Yes |
| rs4782568 | 0.103 | Yes |
| rs4788867 | 0.105 | Yes |
| rs480958 | 0.104 | Yes |
| rs4849147 | 0.104 | Yes |
| rs4865540 | 0.108 | Yes |
| rs4871582 | 0.104 | Yes |
| rs4871827 | 0.103 | Yes |
| rs4876992 | 0.104 | Yes |
| rs4915287 | 0.112 | Yes |
| rs4939034 | 0.107 | Yes |
| rs543874 | 0.106 | Yes |
| rs55665939 | 0.105 | Yes |
| rs55695634 | 0.104 | Yes |
| rs55709272 | 0.111 | Yes |
| rs55824262 | 0.105 | Yes |
| rs55855238 | 0.105 | Yes |
| rs55981844 | 0.105 | Yes |
| rs56015600 | 0.106 | Yes |
| rs56094641 | 0.107 | Yes |
| rs56143801 | 0.106 | Yes |
| rs56189574 | 0.104 | Yes |
| rs56821385 | 0.156 | Yes |
| rs56823429 | 0.102 | Yes |
| rs56960368 | 0.108 | Yes |
| rs57550938 | 0.106 | Yes |
| rs58287327 | 0.104 | Yes |
| rs58542926 | 0.121 | Yes |
| rs58770366 | 0.105 | Yes |
| rs59059615 | 0.106 | Yes |
| rs59316512 | 0.130 | Yes |
| rs59737437 | 0.105 | Yes |
| rs601338 | 0.104 | Yes |
| rs6020459 | 0.104 | Yes |
| rs6072279 | 0.104 | Yes |
| rs6073958 | 0.112 | Yes |
| rs6090103 | 0.110 | Yes |
| rs6138537 | 0.103 | Yes |
| rs613872 | 0.105 | Yes |
| rs61542988 | 0.106 | Yes |
| rs61741874 | 0.116 | Yes |
| rs61812598 | 0.123 | Yes |
| rs61821567 | 0.133 | Yes |
| rs62011286 | 0.105 | Yes |
| rs62092069 | 0.103 | Yes |
| rs62106258 | 0.120 | Yes |
| rs62111724 | 0.102 | Yes |
| rs62118504 | 0.105 | Yes |
| rs62121122 | 0.105 | Yes |
| rs62129471 | 0.104 | Yes |
| rs62158591 | 0.116 | Yes |
| rs62204977 | 0.138 | Yes |
| rs62244890 | 0.103 | Yes |
| rs62282070 | 0.110 | Yes |
| rs623011 | 0.107 | Yes |
| rs62370472 | 0.105 | Yes |
| rs62389532 | 0.122 | Yes |
| rs62451586 | 0.110 | Yes |
| rs62491814 | 0.108 | Yes |
| rs62513191 | 0.120 | Yes |
| rs62618693 | 0.121 | Yes |
| rs6265 | 0.107 | Yes |
| rs6433282 | 0.103 | Yes |
| rs6443429 | 0.102 | Yes |
| rs6445393 | 0.107 | Yes |
| rs6447335 | 0.104 | Yes |
| rs645692 | 0.104 | Yes |
| rs6486122 | 0.108 | Yes |
| rs6501199 | 0.110 | Yes |
| rs6501207 | 0.109 | Yes |
| rs6509155 | 0.105 | Yes |
| rs6509222 | 0.103 | Yes |
| rs6519133 | 0.111 | Yes |
| rs653170 | 0.102 | Yes |
| rs654912 | 0.104 | Yes |
| rs6591188 | 0.104 | Yes |
| rs6595549 | 0.105 | Yes |
| rs6595968 | 0.104 | Yes |
| rs663015 | 0.105 | Yes |
| rs6668050 | 0.129 | Yes |
| rs6698653 | 0.115 | Yes |
| rs6705820 | 0.102 | Yes |
| rs674833 | 0.106 | Yes |
| rs67514550 | 0.115 | Yes |
| rs6786055 | 0.104 | Yes |
| rs6792725 | 0.105 | Yes |
| rs68100606 | 0.104 | Yes |
| rs6816467 | 0.125 | Yes |
| rs684016 | 0.103 | Yes |
| rs6840517 | 0.102 | Yes |
| rs6845703 | 0.103 | Yes |
| rs6905544 | 0.104 | Yes |
| rs6920220 | 0.108 | Yes |
| rs6961634 | 0.107 | Yes |
| rs6962836 | 0.105 | Yes |
| rs6984551 | 0.104 | Yes |
| rs7008413 | 0.117 | Yes |
| rs7012637 | 0.108 | Yes |
| rs704017 | 0.103 | Yes |
| rs7084062 | 0.103 | Yes |
| rs7102088 | 0.105 | Yes |
| rs71322200 | 0.109 | Yes |
| rs71414197 | 0.106 | Yes |
| rs71658797 | 0.113 | Yes |
| rs7171864 | 0.103 | Yes |
| rs7189954 | 0.103 | Yes |
| rs72636644 | 0.111 | Yes |
| rs72654472 | 0.135 | Yes |
| rs72660319 | 0.110 | Yes |
| rs72694393 | 0.105 | Yes |
| rs72732974 | 0.113 | Yes |
| rs72743115 | 0.121 | Yes |
| rs7280982 | 0.108 | Yes |
| rs72837690 | 0.104 | Yes |
| rs728538 | 0.110 | Yes |
| rs72959041 | 0.110 | Yes |
| rs7303035 | 0.103 | Yes |
| rs73137144 | 0.105 | Yes |
| rs7314285 | 0.119 | Yes |
| rs7319102 | 0.105 | Yes |
| rs73201521 | 0.117 | Yes |
| rs73577882 | 0.137 | Yes |
| rs74085345 | 0.165 | Yes |
| rs7430523 | 0.110 | Yes |
| rs74354286 | 0.103 | Yes |
| rs7442885 | 0.102 | Yes |
| rs7488791 | 0.103 | Yes |
| rs74949966 | 0.110 | Yes |
| rs7502409 | 0.103 | Yes |
| rs75064168 | 0.108 | Yes |
| rs7528419 | 0.105 | Yes |
| rs7537072 | 0.103 | Yes |
| rs75460349 | 0.136 | Yes |
| rs75497300 | 0.168 | Yes |
| rs7549881 | 0.103 | Yes |
| rs7551731 | 0.155 | Yes |
| rs75898076 | 0.105 | Yes |
| rs75995782 | 0.120 | Yes |
| rs76102184 | 0.148 | Yes |
| rs76516194 | 0.154 | Yes |
| rs769662 | 0.103 | Yes |
| rs77056528 | 0.149 | Yes |
| rs77101145 | 0.108 | Yes |
| rs77120325 | 0.230 | Yes |
| rs77151304 | 0.123 | Yes |
| rs77243303 | 0.141 | Yes |
| rs77522 | 0.102 | Yes |
| rs77704739 | 0.199 | Yes |
| rs77719426 | 0.227 | Yes |
| rs77808205 | 0.118 | Yes |
| rs77931950 | 0.119 | Yes |
| rs77960347 | 0.261 | Yes |
| rs78343493 | 0.109 | Yes |
| rs78620885 | 0.137 | Yes |
| rs78703482 | 0.116 | Yes |
| rs787488 | 0.104 | Yes |
| rs78769612 | 0.156 | Yes |
| rs78912080 | 0.121 | Yes |
| rs78948780 | 0.206 | Yes |
| rs79101008 | 0.113 | Yes |
| rs7956514 | 0.104 | Yes |
| rs7958316 | 0.140 | Yes |
| rs7970695 | 0.138 | Yes |
| rs79722469 | 0.556 | Yes |
| rs79896703 | 0.109 | Yes |
| rs7993752 | 0.102 | Yes |
| rs8008748 | 0.107 | Yes |
| rs8009347 | 0.103 | Yes |
| rs80272044 | 0.246 | Yes |
| rs8034216 | 0.103 | Yes |
| rs8040040 | 0.106 | Yes |
| rs8054651 | 0.111 | Yes |
| rs8059619 | 0.103 | Yes |
| rs8060025 | 0.103 | Yes |
| rs8077859 | 0.104 | Yes |
| rs8109532 | 0.103 | Yes |
| rs8126001 | 0.103 | Yes |
| rs8178824 | 0.153 | Yes |
| rs846879 | 0.103 | Yes |
| rs855679 | 0.110 | Yes |
| rs8978 | 0.103 | Yes |
| rs9266230 | 0.107 | Yes |
| rs9366639 | 0.110 | Yes |
| rs9368503 | 0.103 | Yes |
| rs9383643 | 0.104 | Yes |
| rs9388766 | 0.109 | Yes |
| rs9611454 | 0.106 | Yes |
| rs9738365 | 0.105 | Yes |
| rs9788721 | 0.102 | Yes |
| rs9826984 | 0.103 | Yes |
| rs984181 | 0.139 | Yes |
| rs9929143 | 0.114 | Yes |
| rs9951447 | 0.102 | Yes |
| rs9974178 | 0.104 | Yes |
| rs9988620 | 0.106 | Yes |
| rs10521222 | 0.137 | Yes |
| rs1183910 | 0.142 | Yes |
| rs12239046 | 0.106 | Yes |
| rs2794520 | 0.155 | Yes |
| rs2847281 | 0.103 | Yes |
| rs4129267 | 0.123 | Yes |
| rs4420065 | 0.143 | Yes |
| rs4705952 | 0.105 | Yes |
| rs6734238 | 0.113 | Yes |
| rs6901250 | 0.102 | Yes |
| Allele score | 1.532 | No |

| **Supplementary Table 11: Results of the instrumental inequalities for MR models for the effect of triglyceride concentrations on coronary artery disease in pseudo populations inverse probability weighted for 10 principal components** | | |
| --- | --- | --- |
| **Proposed instrument** | **Value of instrumental inequalities** | **Do instrumental inequalities hold?** |
| rs38855 | 0.104 | Yes |
| rs3198697 | 0.104 | Yes |
| rs8077889 | 0.104 | Yes |
| rs10493326 | 0.112 | Yes |
| rs10513688 | 0.110 | Yes |
| rs10029254 | 0.107 | Yes |
| rs799160 | 0.108 | Yes |
| rs9693857 | 0.104 | Yes |
| rs4921914 | 0.112 | Yes |
| rs7033354 | 0.104 | Yes |
| rs1781930 | 0.104 | Yes |
| rs603446 | 0.111 | Yes |
| rs10861661 | 0.108 | Yes |
| rs1341267 | 0.103 | Yes |
| rs1035744 | 0.104 | Yes |
| rs749671 | 0.102 | Yes |
| rs9930333 | 0.102 | Yes |
| rs1688030 | 0.123 | Yes |
| rs3761445 | 0.105 | Yes |
| rs719726 | 0.106 | Yes |
| rs1495741 | 0.113 | Yes |
| rs7248104 | 0.103 | Yes |
| Allele score | 1.021 | *No* |

| **Supplementary Table 12: Results of the instrumental inequalities for MR models for the effect of HDL-cholesterol on coronary artery disease in pseudo populations inverse-probability weighted for 10 principal components** | | |
| --- | --- | --- |
| **Proposed instrument** | **Value of instrumental inequalities** | **Do instrumental inequalities hold?** |
| rs12145743 | 0.102 | Yes |
| rs4650994 | 0.104 | Yes |
| rs13326165 | 0.104 | Yes |
| rs2602836 | 0.103 | Yes |
| rs4917014 | 0.107 | Yes |
| rs17173637 | 0.106 | Yes |
| rs970548 | 0.109 | Yes |
| rs2923084 | 0.106 | Yes |
| rs12801636 | 0.106 | Yes |
| rs499974 | 0.111 | Yes |
| rs4983559 | 0.106 | Yes |
| rs16942887 | 0.117 | Yes |
| rs17695224 | 0.104 | Yes |
| rs181362 | 0.108 | Yes |
| rs4660293 | 0.112 | Yes |
| rs13107325 | 0.133 | Yes |
| rs6450176 | 0.106 | Yes |
| rs702485 | 0.104 | Yes |
| rs2293889 | 0.107 | Yes |
| rs11869286 | 0.108 | Yes |
| rs1689800 | 0.105 | Yes |
| rs4759375 | 0.112 | Yes |
| rs581080 | 0.110 | Yes |
| rs605066 | 0.105 | Yes |
| rs7255436 | 0.106 | Yes |
| rs737337 | 0.133 | Yes |
| rs838880 | 0.108 | Yes |
| rs12133576 | 0.105 | Yes |
| rs1689797 | 0.106 | Yes |
| rs6680658 | 0.104 | Yes |
| rs355838 | 0.105 | Yes |
| rs1047891 | 0.103 | Yes |
| rs2290547 | 0.108 | Yes |
| rs2240327 | 0.104 | Yes |
| rs6805251 | 0.103 | Yes |
| rs1482852 | 0.104 | Yes |
| rs10019888 | 0.107 | Yes |
| rs3822072 | 0.105 | Yes |
| rs4976033 | 0.102 | Yes |
| rs205262 | 0.108 | Yes |
| rs9491696 | 0.104 | Yes |
| rs12525163 | 0.102 | Yes |
| rs17286602 | 0.102 | Yes |
| rs10282707 | 0.106 | Yes |
| rs3996352 | 0.106 | Yes |
| rs4332136 | 0.205 | Yes |
| rs4871137 | 0.106 | Yes |
| rs4075205 | 0.105 | Yes |
| rs686030 | 0.113 | Yes |
| rs2472509 | 0.104 | Yes |
| rs2303975 | 0.109 | Yes |
| rs17788930 | 0.108 | Yes |
| rs11246602 | 0.115 | Yes |
| rs12226802 | 0.114 | Yes |
| rs7117842 | 0.104 | Yes |
| rs11045163 | 0.104 | Yes |
| rs2241210 | 0.105 | Yes |
| rs838876 | 0.109 | Yes |
| rs10773105 | 0.106 | Yes |
| rs931992 | 0.108 | Yes |
| rs4148005 | 0.103 | Yes |
| rs4969178 | 0.109 | Yes |
| rs4939883 | 0.124 | Yes |
| rs11660468 | 0.110 | Yes |
| rs952044 | 0.106 | Yes |
| rs2278236 | 0.106 | Yes |
| rs103294 | 0.110 | Yes |
| rs1121980 | 0.107 | Yes |
| rs12328675 | 0.117 | Yes |
| rs2954029 | 0.107 | Yes |
| rs3136441 | 0.116 | Yes |
| rs386000 | 0.109 | Yes |
| rs7134375 | 0.105 | Yes |
| rs7941030 | 0.105 | Yes |
| rs2606736 | 0.102 | Yes |
| rs4129767 | 0.107 | Yes |
| rs4731702 | 0.106 | Yes |
| rs4765127 | 0.109 | Yes |
| rs7134594 | 0.105 | Yes |
| rs1936800 | 0.104 | Yes |
| rs4142995 | 0.106 | Yes |
| rs4148008 | 0.104 | Yes |
| Allele score | 1.061 | *No* |

| **Supplemental Table 13: Results of the instrumental inequalities applied to MR models for the effect of LDL-cholesterol on coronary artery disease in pseudo populations inverse-probability weighted for 10 principal components** | | |
| --- | --- | --- |
| **Proposed instrument** | **Value of instrumental inequalities** | **Do instrumental inequalities hold?** |
| rs10027182 | 0.105 | Yes |
| rs267733 | 0.108 | Yes |
| rs11563251 | 0.106 | Yes |
| rs7640978 | 0.113 | Yes |
| rs1564348 | 0.117 | Yes |
| rs12670798 | 0.112 | Yes |
| rs10102164 | 0.108 | Yes |
| rs3780181 | 0.113 | Yes |
| rs1169288 | 0.109 | Yes |
| rs4942486 | 0.106 | Yes |
| rs8017377 | 0.106 | Yes |
| rs364585 | 0.102 | Yes |
| rs2328223 | 0.109 | Yes |
| rs5763662 | 0.128 | Yes |
| rs10903129 | 0.105 | Yes |
| rs2587534 | 0.108 | Yes |
| rs515135 | 0.123 | Yes |
| rs6544713 | 0.119 | Yes |
| rs4148218 | 0.110 | Yes |
| rs2710642 | 0.104 | Yes |
| rs17508045 | 0.116 | Yes |
| rs2030746 | 0.104 | Yes |
| rs16831243 | 0.112 | Yes |
| rs1250229 | 0.104 | Yes |
| rs9875338 | 0.103 | Yes |
| rs4530754 | 0.105 | Yes |
| rs1800562 | 0.148 | Yes |
| rs2297374 | 0.105 | Yes |
| rs2073547 | 0.113 | Yes |
| rs217386 | 0.104 | Yes |
| rs2737252 | 0.105 | Yes |
| rs7832643 | 0.105 | Yes |
| rs8176720 | 0.103 | Yes |
| rs579459 | 0.112 | Yes |
| rs10832962 | 0.104 | Yes |
| rs11220462 | 0.118 | Yes |
| rs2000999 | 0.120 | Yes |
| rs314253 | 0.104 | Yes |
| rs6511720 | 0.141 | Yes |
| rs688 | 0.109 | Yes |
| rs6859 | 0.115 | Yes |
| rs492602 | 0.110 | Yes |
| rs7264396 | 0.104 | Yes |
| rs6016381 | 0.105 | Yes |
| rs6065311 | 0.106 | Yes |
| rs2479409 | 0.110 | Yes |
| rs2642442 | 0.104 | Yes |
| rs629301 | 0.125 | Yes |
| rs4299376 | 0.119 | Yes |
| rs6818397 | 0.103 | Yes |
| rs3177928 | 0.114 | Yes |
| rs3757354 | 0.106 | Yes |
| rs11136341 | 0.105 | Yes |
| rs1801689 | 0.121 | Yes |
| rs1998013 | 0.467 | Yes |
| rs6603981 | 0.104 | Yes |
| rs903319 | 0.104 | Yes |
| rs2287623 | 0.103 | Yes |
| rs17345563 | 0.110 | Yes |
| rs7703051 | 0.115 | Yes |
| rs2294261 | 0.105 | Yes |
| rs17789218 | 0.106 | Yes |
| rs868943 | 0.103 | Yes |
| rs2326077 | 0.108 | Yes |
| rs174532 | 0.109 | Yes |
| rs6489818 | 0.110 | Yes |
| rs1186380 | 0.105 | Yes |
| rs2288002 | 0.104 | Yes |
| rs4791641 | 0.103 | Yes |
| rs7225700 | 0.105 | Yes |
| rs10410 | 0.111 | Yes |
| rs10910490 | 0.107 | Yes |
| rs11102964 | 0.110 | Yes |
| rs11206510 | 0.114 | Yes |
| rs11206514 | 0.108 | Yes |
| rs11485618 | 0.107 | Yes |
| rs11581665 | 0.110 | Yes |
| rs11583974 | 0.128 | Yes |
| rs11591147 | 0.477 | Yes |
| rs12066643 | 0.104 | Yes |
| rs12127701 | 0.119 | Yes |
| rs12129277 | 0.107 | Yes |
| rs12410656 | 0.117 | Yes |
| rs1278286 | 0.110 | Yes |
| rs1337247 | 0.108 | Yes |
| rs13375691 | 0.110 | Yes |
| rs1386585 | 0.103 | Yes |
| rs1475701 | 0.125 | Yes |
| rs17035630 | 0.111 | Yes |
| rs17035665 | 0.108 | Yes |
| rs17647543 | 0.124 | Yes |
| rs1874776 | 0.106 | Yes |
| rs207145 | 0.106 | Yes |
| rs2247213 | 0.104 | Yes |
| rs2479394 | 0.108 | Yes |
| rs2647281 | 0.110 | Yes |
| rs413380 | 0.130 | Yes |
| rs413582 | 0.107 | Yes |
| rs4847221 | 0.108 | Yes |
| rs4927207 | 0.111 | Yes |
| rs4970712 | 0.104 | Yes |
| rs572512 | 0.104 | Yes |
| rs585131 | 0.107 | Yes |
| rs585362 | 0.116 | Yes |
| rs650985 | 0.117 | Yes |
| rs6662286 | 0.113 | Yes |
| rs6689614 | 0.111 | Yes |
| rs72703204 | 0.155 | Yes |
| rs7512480 | 0.104 | Yes |
| rs7544735 | 0.104 | Yes |
| rs7552841 | 0.107 | Yes |
| rs10195252 | 0.103 | Yes |
| rs10208987 | 0.111 | Yes |
| rs1025447 | 0.107 | Yes |
| rs10490626 | 0.115 | Yes |
| rs11096689 | 0.114 | Yes |
| rs11679386 | 0.115 | Yes |
| rs11685356 | 0.117 | Yes |
| rs12471982 | 0.114 | Yes |
| rs12691202 | 0.136 | Yes |
| rs12720796 | 0.126 | Yes |
| rs12720842 | 0.134 | Yes |
| rs13027175 | 0.131 | Yes |
| rs1534420 | 0.104 | Yes |
| rs17398765 | 0.122 | Yes |
| rs2194562 | 0.105 | Yes |
| rs312049 | 0.109 | Yes |
| rs3791981 | 0.118 | Yes |
| rs4148177 | 0.107 | Yes |
| rs492399 | 0.118 | Yes |
| rs4953023 | 0.127 | Yes |
| rs4988235 | 0.108 | Yes |
| rs6413458 | 0.293 | Yes |
| rs6547409 | 0.137 | Yes |
| rs6725189 | 0.110 | Yes |
| rs6729410 | 0.106 | Yes |
| rs6739502 | 0.107 | Yes |
| rs6754295 | 0.112 | Yes |
| rs6756743 | 0.143 | Yes |
| rs6759321 | 0.106 | Yes |
| rs75279593 | 0.123 | Yes |
| rs7567653 | 0.130 | Yes |
| rs780093 | 0.111 | Yes |
| rs11709504 | 0.106 | Yes |
| rs17819328 | 0.102 | Yes |
| rs10069744 | 0.113 | Yes |
| rs10515198 | 0.120 | Yes |
| rs10515214 | 0.112 | Yes |
| rs12916 | 0.115 | Yes |
| rs16872670 | 0.113 | Yes |
| rs3857388 | 0.113 | Yes |
| rs4361493 | 0.111 | Yes |
| rs4703642 | 0.105 | Yes |
| rs4704231 | 0.111 | Yes |
| rs4704810 | 0.103 | Yes |
| rs6873053 | 0.117 | Yes |
| rs7717505 | 0.117 | Yes |
| rs7727150 | 0.104 | Yes |
| rs10455872 | 0.121 | Yes |
| rs117733303 | 0.164 | Yes |
| rs12208357 | 0.129 | Yes |
| rs1367211 | 0.106 | Yes |
| rs1408272 | 0.143 | Yes |
| rs16891156 | 0.164 | Yes |
| rs2327951 | 0.104 | Yes |
| rs2621321 | 0.110 | Yes |
| rs3120139 | 0.110 | Yes |
| rs3798180 | 0.106 | Yes |
| rs3798221 | 0.108 | Yes |
| rs389883 | 0.106 | Yes |
| rs3918291 | 0.711 | Yes |
| rs446218 | 0.105 | Yes |
| rs461473 | 0.112 | Yes |
| rs6909746 | 0.103 | Yes |
| rs6917747 | 0.112 | Yes |
| rs6935921 | 0.105 | Yes |
| rs7774197 | 0.124 | Yes |
| rs9457843 | 0.110 | Yes |
| rs6461566 | 0.106 | Yes |
| rs10102352 | 0.105 | Yes |
| rs13277801 | 0.108 | Yes |
| rs2081687 | 0.108 | Yes |
| rs2980875 | 0.113 | Yes |
| rs4360309 | 0.107 | Yes |
| rs4592055 | 0.111 | Yes |
| rs11244084 | 0.120 | Yes |
| rs11795315 | 0.106 | Yes |
| rs4489379 | 0.103 | Yes |
| rs630014 | 0.103 | Yes |
| rs7030248 | 0.104 | Yes |
| rs8176693 | 0.117 | Yes |
| rs2419604 | 0.104 | Yes |
| rs10893493 | 0.105 | Yes |
| rs10893499 | 0.114 | Yes |
| rs10893505 | 0.117 | Yes |
| rs11600380 | 0.109 | Yes |
| rs12294259 | 0.123 | Yes |
| rs174476 | 0.106 | Yes |
| rs174583 | 0.106 | Yes |
| rs180326 | 0.107 | Yes |
| rs2075290 | 0.124 | Yes |
| rs2845573 | 0.116 | Yes |
| rs508487 | 0.127 | Yes |
| rs582037 | 0.109 | Yes |
| rs10850003 | 0.110 | Yes |
| rs11065987 | 0.103 | Yes |
| rs11066028 | 0.107 | Yes |
| rs17630235 | 0.103 | Yes |
| rs2708101 | 0.103 | Yes |
| rs3184504 | 0.104 | Yes |
| rs657197 | 0.107 | Yes |
| rs7953150 | 0.106 | Yes |
| rs12448528 | 0.105 | Yes |
| rs12931964 | 0.110 | Yes |
| rs1864163 | 0.107 | Yes |
| rs217181 | 0.108 | Yes |
| rs247616 | 0.109 | Yes |
| rs7197967 | 0.107 | Yes |
| rs8044335 | 0.105 | Yes |
| rs8044476 | 0.109 | Yes |
| rs8060878 | 0.105 | Yes |
| rs9302635 | 0.109 | Yes |
| rs2886232 | 0.120 | Yes |
| rs6504872 | 0.105 | Yes |
| rs7206971 | 0.105 | Yes |
| rs8070463 | 0.104 | Yes |
| rs10402271 | 0.119 | Yes |
| rs10403668 | 0.109 | Yes |
| rs10419669 | 0.107 | Yes |
| rs10422616 | 0.103 | Yes |
| rs10460181 | 0.115 | Yes |
| rs1048699 | 0.126 | Yes |
| rs11668536 | 0.105 | Yes |
| rs11669133 | 0.124 | Yes |
| rs11881156 | 0.113 | Yes |
| rs12150984 | 0.105 | Yes |
| rs12721109 | 0.343 | Yes |
| rs1529711 | 0.108 | Yes |
| rs1531517 | 0.201 | Yes |
| rs157580 | 0.118 | Yes |
| rs1594895 | 0.108 | Yes |
| rs16979372 | 0.161 | Yes |
| rs16996148 | 0.167 | Yes |
| rs17677316 | 0.110 | Yes |
| rs17800760 | 0.117 | Yes |
| rs1799898 | 0.106 | Yes |
| rs2075650 | 0.154 | Yes |
| rs2287019 | 0.105 | Yes |
| rs2315025 | 0.105 | Yes |
| rs2927477 | 0.121 | Yes |
| rs2965101 | 0.119 | Yes |
| rs2965157 | 0.232 | Yes |
| rs2965174 | 0.107 | Yes |
| rs2972564 | 0.111 | Yes |
| rs3208856 | 0.212 | Yes |
| rs36005514 | 0.125 | Yes |
| rs376642 | 0.104 | Yes |
| rs3786721 | 0.106 | Yes |
| rs3786722 | 0.110 | Yes |
| rs379309 | 0.105 | Yes |
| rs387976 | 0.116 | Yes |
| rs445925 | 0.309 | Yes |
| rs4803760 | 0.141 | Yes |
| rs4803766 | 0.106 | Yes |
| rs5158 | 0.115 | Yes |
| rs5742911 | 0.110 | Yes |
| rs59325138 | 0.112 | Yes |
| rs714948 | 0.116 | Yes |
| rs7251031 | 0.109 | Yes |
| rs7252981 | 0.105 | Yes |
| rs7255743 | 0.205 | Yes |
| rs73015030 | 0.155 | Yes |
| rs73045960 | 0.950 | Yes |
| rs77301115 | 0.156 | Yes |
| rs8102380 | 0.102 | Yes |
| rs8103315 | 0.113 | Yes |
| rs8108762 | 0.109 | Yes |
| rs892114 | 0.113 | Yes |
| rs926054 | 0.118 | Yes |
| rs9973305 | 0.120 | Yes |
| rs2745865 | 0.109 | Yes |
| rs2865507 | 0.104 | Yes |
| rs2902940 | 0.105 | Yes |
| rs4142393 | 0.103 | Yes |
| rs4812494 | 0.106 | Yes |
| rs6124309 | 0.106 | Yes |
| rs742748 | 0.104 | Yes |
| rs4253772 | 0.108 | Yes |
| rs4253776 | 0.105 | Yes |
| rs76733602 | 0.118 | Yes |
| rs68160747 | 0.126 | Yes |
| rs72768351 | 0.155 | Yes |
| rs4703665 | 0.108 | Yes |
| rs6877840 | 0.115 | Yes |
| rs17244939 | 1.004 | Yes |
| rs5908 | 0.140 | Yes |
| rs74695562 | 0.115 | Yes |
| rs7706933 | 0.140 | Yes |
| rs3804231 | 0.120 | Yes |
| rs80324692 | 0.117 | Yes |
| rs75944831 | 0.155 | Yes |
| rs62366598 | 0.110 | Yes |
| rs11206479 | 0.102 | Yes |
| rs10218716 | 0.103 | Yes |
| rs2500340 | 0.104 | Yes |
| rs943645 | 0.102 | Yes |
| rs12093385 | 0.106 | Yes |
| rs12075602 | 0.105 | Yes |
| rs17111474 | 0.102 | Yes |
| rs2479398 | 0.105 | Yes |
| rs2864123 | 0.125 | Yes |
| rs2479396 | 0.104 | Yes |
| rs17111483 | 0.112 | Yes |
| rs17111490 | 0.108 | Yes |
| rs12739979 | 0.107 | Yes |
| rs11810371 | 0.120 | Yes |
| rs77406753 | 0.104 | Yes |
| rs2479406 | 0.107 | Yes |
| rs28385701 | 0.191 | Yes |
| rs74700387 | 0.174 | Yes |
| rs28385708 | 0.126 | Yes |
| rs11800243 | 0.120 | Yes |
| rs12067569 | 0.131 | Yes |
| rs10493176 | 0.125 | Yes |
| rs17111657 | 0.118 | Yes |
| rs960502 | 0.372 | Yes |
| rs12138592 | 0.117 | Yes |
| rs80355736 | 0.167 | Yes |
| rs117175530 | 0.152 | Yes |
| rs4724302 | 0.116 | Yes |
| rs73107405 | 0.126 | Yes |
| rs113496141 | 0.117 | Yes |
| rs6956388 | 0.119 | Yes |
| rs10234070 | 0.109 | Yes |
| rs76236858 | 0.218 | Yes |
| rs76331929 | 0.129 | Yes |
| rs217437 | 0.102 | Yes |
| rs35349497 | 0.121 | Yes |
| rs41279627 | 0.128 | Yes |
| rs113223818 | 0.135 | Yes |
| rs56243746 | 0.127 | Yes |
| rs117843715 | 0.142 | Yes |
| rs117623941 | 0.155 | Yes |
| rs2008036 | 0.110 | Yes |
| rs77516609 | 0.115 | Yes |
| rs7792931 | 0.133 | Yes |
| rs118187778 | 0.123 | Yes |
| rs79836087 | 0.127 | Yes |
| rs76446485 | 0.143 | Yes |
| rs80169634 | 0.118 | Yes |
| rs76203970 | 0.212 | Yes |
| rs114776298 | 0.117 | Yes |
| rs1801695 | 0.118 | Yes |
| rs1801700 | 0.117 | Yes |
| rs72653053 | 0.195 | Yes |
| rs62122522 | 0.124 | Yes |
| rs114368165 | 0.187 | Yes |
| rs115071638 | 0.154 | Yes |
| rs12470778 | 0.114 | Yes |
| rs312966 | 0.103 | Yes |
| rs113588790 | 0.119 | Yes |
| rs72782175 | 0.278 | Yes |
| rs1801703 | 0.213 | Yes |
| rs1801699 | 0.166 | Yes |
| rs79112951 | 0.113 | Yes |
| rs17519079 | 0.113 | Yes |
| rs1263149 | 0.102 | Yes |
| rs34552724 | 0.144 | Yes |
| rs78296522 | 0.123 | Yes |
| rs74773964 | 0.140 | Yes |
| rs633389 | 0.153 | Yes |
| rs5110 | 0.118 | Yes |
| rs11216164 | 0.104 | Yes |
| rs548638 | 0.104 | Yes |
| rs11216169 | 0.107 | Yes |
| rs543819 | 0.114 | Yes |
| rs17174502 | 0.114 | Yes |
| rs7943309 | 0.131 | Yes |
| rs7106782 | 0.119 | Yes |
| rs17678136 | 0.112 | Yes |
| rs13345127 | 0.113 | Yes |
| rs2072382 | 0.127 | Yes |
| rs2421198 | 0.103 | Yes |
| rs17242346 | 0.130 | Yes |
| rs12981050 | 0.119 | Yes |
| rs17242367 | 0.116 | Yes |
| rs17248748 | 0.211 | Yes |
| rs2569556 | 0.119 | Yes |
| rs6413503 | 0.153 | Yes |
| rs3826810 | 0.114 | Yes |
| rs72658879 | 0.125 | Yes |
| rs4804146 | 0.105 | Yes |
| rs892113 | 0.124 | Yes |
| rs4804149 | 0.105 | Yes |
| rs11880059 | 0.117 | Yes |
| rs17616661 | 0.109 | Yes |
| rs4804150 | 0.103 | Yes |
| rs4804576 | 0.128 | Yes |
| rs12027135 | 0.105 | Yes |
| rs514230 | 0.107 | Yes |
| rs2072183 | 0.112 | Yes |
| rs6029526 | 0.106 | Yes |
| Allele score | 1.493 | *No* |

| **Supplementary table 14: Comparison of Instrumental inequalities, MR Egger intercept test and MR-PRESSO global test to detect violations of IV assumptions when all proposed instruments are used jointly** | | | | | | | | |
| --- | --- | --- | --- | --- | --- | --- | --- | --- |
| Exposure | Violation of IV inequalities for SNPs jointly | Maximum value of the inequalities for SNPs jointly | Violation of IV conditions indicated by MR-Egger intercept test for SNPs jointly | Estimated value of MR-Egger intercept | P-value associated with the MR-Egger intercept test | Violations of IV conditions indicated by MR-PRESSO global test for SNPs jointly | P-value associated with MR-PRESSO global test |  |
| Vitamin D | Yes | 2.000 | No | 3.588 x 10^-5 | 0.914 | No | 0.941 |  |
| Alcohol consumption | Yes | 2.000 | No | 3.427 x 10^-4 | 0.081 | Yes | <0.001 |  |
| CRP | Yes | 2.000 | Yes | 2.971 x 10^-4 | <0.001 | Yes | <0.001 |  |
| Triglycerides | Yes | 2.000 | Yes | 8.931 x 10^-4 | <0.001 | Yes | <0.001 |  |
| HDL-cholesterol | Yes | 2.000 | Yes | -3.474 x 10^-4 | 0.018 | Yes | <0.001 |  |
| LDL-cholesterol | Yes | 2.000 | No | 5.560 x 10^-5 | 0.233 | Yes | <0.001 |  |

**S10. Supplementary table 14: Results of the instrumental inequalities, MR-Egger intercept test and MR-PRESSO global test for SNPs proposed jointly as instruments**

**S11. R Code: Simulating a toy example for the instrumental inequalities**

*# R Code simulating a toy example for the instrumental inequalities*

*# Based on R code created by Diemer et al. 2020*

*# Created by: K. Guo, E.w. Diemer, J.A. Labrecque, S.A. Swanson*

*# Last edited: 09/11/2022*

run_instrumental_inequalities_singlejointiv <- **function**(data,

y,

x,

instrument,

weight = NULL,

tables_out = FALSE){

**library**(dplyr)

**library**(boot)

*# Set variable names*

data$Y <- data[[y]]

data$X <- data[[x]]

data$IV <- data[[instrument]]

**if**(is.null(weight)){data$weight <- 1}**else**{data$weight <- data[[weight]]}

*# Count the frequencies of the different values for IV*

IV_counts <- data %>%

group_by(IV) %>%

tally(., wt = weight) %>%

rename(n_IV=n)

*# Count the frequencies of the different combinations for IV, X and Y*

grouped_counts <- data %>%

group_by(IV, X, Y) %>%

tally(., wt = weight)

*# Merge counts and fill na with 0*

grouped_counts <- merge(grouped_counts, IV_counts, by='IV')

grouped_counts$n_IV[is.na(grouped_counts$n_IV)] <- 0

*# Calculate proportions*

grouped_counts$p = grouped_counts$n / grouped_counts$n_IV

*# Calculate the max per X, Y (over IV), sum per X (over Y), calculate max*

*# Equation [3] Pearl, J. (1995)*

pearl <- grouped_counts %>%

group_by(X, Y) %>%

summarise(max_p = max(p)) %>%

group_by(X) %>%

summarise(sum_maxp=sum(max_p)) %>% suppressMessages()

max_ineq <- max(pearl$sum_maxp)

**if** (tables_out) {

**return**(list(max_ineq = max_ineq,

grouped_counts = grouped_counts,

max_xy = grouped_counts %>%

group_by(X, Y) %>%

summarise(max_p = max(p)) %>% suppressMessages() %>% as.data.frame,

sum_x = pearl %>% as.data.frame())

)

} **else**{

**return**(max_ineq)

}

}

sim <- **function**(n = 1e5,

z_prev =0.5,

z_size = 1,

int_x = 0.2,

int_y = 0.2,

or_zx = 2,

or_xy = 2,

or_zy = 1,

tables_out = FALSE,

data_out = FALSE,

seed = NULL) {

**library**(boot)

*# Sets the seed for reproducible results*

**if**(!is.null(seed)) {

set.seed(seed)

}

*# Simulate random binomial distributed data according some input of association*

*# between outcome y, exposure x and instrument z*

z <- rbinom(n,z_size,prob = z_prev)

x <- rbinom(n, 1, prob = inv.logit(log(int_x/(1-int_x)) + log(or_zx)*z))

y <- rbinom(n, 1, prob = inv.logit(log(int_y/(1-int_y)) + log(or_xy)*x + log(or_zy)*z))

ds <- data.frame(z,x,y)

out <- run_instrumental_inequalities_singlejointiv(data = ds, y = "y", x = "x", instrument = "z" , tables_out = tables_out)

**if** (data_out) {

**return**(list(data_out = ds,

ineq_out = out))

} **else** {

**return**(ineq_out = out)

}

}

*# TABLES FOR TOY EXAMPLE*

*# No pleiotropy*

sim(n = 1e5, tables_out = TRUE, data_out = TRUE, z_size = 2, seed = 26)

sim(n = 1e5, tables_out = TRUE, data_out = TRUE, z_size = 2 , or_zy = 5, seed = 26)

**S12. R Code: Computing the maximum value of the instrumental inequalities for a single joint instrument**

*# Function to get maximum value of the instrumental inequalities for single joint instrument*

*# Based on R code created by Diemer et al. 2020*

*# Created by: K. Guo, E.w. Diemer, J.A. Labrecque, S.A. Swanson*

*# Last edited: 29/03/2022*

*# install.packages("tidyverse")*

**library**(tidyverse)

run_instrumental_inequalities_singlejointiv <- **function**(data.

y.

x.

instrument.

weight = NULL){

*# # Set variable names*

data$Y <- data[[y]]

data$X <- data[[x]]

data$IV <- data[[instrument]]

**if**(is.null(weight)){data$weight <- 1}**else**{data$weight <- data[[weight]]}

*# Count the frequencies of the different values for IV*

IV_counts <- data %>%

group_by(IV) %>%

tally(.. wt = weight) %>%

rename(n_IV=n)

*# Count the frequencies of the different combinations for IV. X and Y*

grouped_counts <- data %>%

group_by(IV. X. Y) %>%

tally(.. wt = weight)

*# Merge counts and fill na with 0*

grouped_counts <- merge(grouped_counts. IV_counts. by='IV')

grouped_counts$n_IV[is.na(grouped_counts$n_IV)] <- 0

*# Calculate proportions*

grouped_counts$p = grouped_counts$n / grouped_counts$n_IV

*# Calculate the max per X. Y (over IV). sum per X (over Y). calculate max*

*# Equation [3] Pearl. J. (1995)*

pearl <- grouped_counts %>%

group_by(X. Y) %>%

summarise(max_p = max(p)) %>%

group_by(X) %>%

summarise(sum_maxp=sum(max_p))

max_ineq <- max(pearl$sum_maxp)

**return**(max_ineq)

}
